# Composition of Proteins Associated with Red Clover (*Trifolium pratense*) and the Microbiota Identified in Honey

**DOI:** 10.3390/life14070862

**Published:** 2024-07-10

**Authors:** Violeta Čeksterytė, Algirdas Kaupinis, Andrius Aleliūnas, Rūta Navakauskienė, Kristina Jaškūnė

**Affiliations:** 1 LAMMC—Institute of Agriculture, Lithuanian Research Centre for Agriculture and Forestry, Instituto Ave. 1, 58344 Akademija, Lithuania; violeta.ceksteryte@lammc.lt (V.Č.); andrius.aleliunas@lammc.lt (A.A.); 2 VU GMC—Life Sciences Center, Vilnius University, Saulėtekio Ave. 7, 10257 Vilnius, Lithuania; algirdas.kaupinis@gf.vu.lt (A.K.); ruta.navakauskiene@bchi.vu.lt (R.N.)

**Keywords:** aphid microbiota, biological process, cellular component, clover honey, molecular function, lactic acid bacteria

## Abstract

The nutritional composition of honey is determined by environmental conditions, and botanical and geographical origin. In addition to carbohydrates, honey also contain pollen grains, proteins, free amino acids, and minerals. Although the content of proteins in honey is low, they are an important component that confirms the authenticity and quality of honey; therefore, they became a popular study object. The aim of the study was to evaluate protein content and composition of monofloral red clover and rapeseed honey collected from five different districts of Lithuania. Forty-eight proteins were identified in five different origin honey samples by liquid chromatography. The number of red clover proteins identified in individual honey samples in monofloral red clover honey C3 was 39 in polyfloral honey S22–36, while in monofloral rapeseed honey S5, S15, and S23 there was 33, 32, and 40 respectively. Aphids’ proteins and lactic acid bacteria were identified in all honey samples tested. The linear relationship and the strongest correlation coefficient (r = 0.97) were determined between the content of *Apilactobacillus kunkeei* and *Apilactobacillus apinorum*, as well as between the number of faba bean (*Vicia faba*) pollen and lactic acid bacteria (r = 0.943). The data show a strong correlation coefficient between the amount of lactic acid and aphid protein number (r = 0.693). More studies are needed to evaluate the relationship between the pollination efficiency of red clover by bees and the multiplicity of red clover proteins in honey protein, as well as microbiota diversity and the influence of nature or plant diversity on the occurrence of microbiota in honey.

## 1. Introduction

The most suitable plant pollinators are honeybees due to their biology and adaptability to plants [1]. Some plant species are selectively pollinated by a single insect species, and this has a significant effect on plant seed productivity and survival [2]. The flowers of red clover have a long, approximately 10 mm, corolla tube and nectarines are located at nectary base of it, making the nectar collection difficult for short-tongued bees [3]. The most important pollinators of red clover belong to different species and subspecies of wild long-tongued bumblebees (*Bombus* ssp.), such as *B. pascuorum* ssp., *B. ruderatus*, and *B. hortorum* ssp., and some races or hybrids of honeybees [3,4]. While long-tongued bumblebees collect nectar by reaching it down in the corolla tube, short-tongued bumblebees, such as *B. terrestris* and *B. lucorum*, bite holes in the lower part of the corolla to access nectar without pollinating, thus reducing pollination efficiency and seed yield [5]. Bees can also bite holes in corolla tubes or use the holes previously bitten by bumblebees to collect nectar from red clover, resulting in no pollen transfer to nectar [6]. The study of diploid and tetraploid clover seed production revealed that tetraploid varieties produce more nectar per floret than diploid ones; however, that differences in corolla tube dimensions due to ploidy level did not affect seed production, suggesting that insects visited and pollinated the flowers [7]. Therefore, it is necessary to maintain medium- and long-tongued honeybee populations and to protect the variety of wild pollinators [8]. The studies of Balžekas et al. [4,9] reveal that Caucasian x European dark bee (*Apis mellifera mellifera* L. have been widespread in Lithuania since ancient times, and are listed as a Lithuanian native bee) hybrids and Caucasian × Carniolan bee hybrids collected 74.8% and 65.6% more honey per colony compared to pure Caucasian bees, respectively.

The earlier studies on the protein composition of manually collected clover pollen from flowers of red clover cvs. ‘Kiršiniai’ and ‘Vyčiai’, berseem clover (*Trifolium alexandrinum* L.) cv. ‘Faraon’, and white clover (*Trifolium repens* L.) cv. ‘Medūnai’ identified and described over 200 protein spots from which quantitative levels were most divergent in 30 investigated clover pollen proteome maps [10,11]. The berseem clover honey had no sulfur-containing amino acids methionine and cysteine, but contained a sufficiently high amount of lysine and was the most acidic (pH 3.26) compared to the other types of honey studied [12]. It is known that legumes possess antioxidant enzymes, such as superoxide dismutase, catalase, and various peroxidases as well as non-enzymatic antioxidants such as ascorbate and glutathione, which protect them from reactive oxygen species (ROS) and have a symbiotic relationship with nitrogen-fixing soil bacteria called rhizobia [13,14]. Legumes have been shown to have various proteins, such as natural resistance-associated macrophage proteins/duodenal metal transporter (NRAMP/DMT) homologs involved in metal ion transport across membranes within the legume nodule [15], transferrin-mediating iron transport [16], and comprise about 24% mass of leghaemoglobin present in legume nodules [17]. Another protein identified in legumes that contains iron is ferritin, whose accumulation (around 24 dpi) is correlated with the highest level of leghaemoglobin [18]. Ferritin constitutes almost half of the of total seed iron in soybeans, common beans, and peas and, depending on the plant species, can range from 18 to 42% and is known for participating in cell detoxification as well as indicating stress-induced responses [19,20]. Therefore, the identification of legume proteins and the determination of their role in various biological processes are of great importance.

The composition of sugars and proteins in nectar honey differs from that of honeydew honey in terms of color, organoleptic properties, and the values for electrical conductivity, pH, optical rotation, ash content, sugar profile, and mineral content; however, physicochemical indicators do not necessarily reflect its authenticity [21]. For better identification chromatography, spectroscopy, and molecular biology approaches are used along with melissopalynological analysis when the botanical origin of honey pollen and the amount of honeydew elements are visually assessed [22,23,24,25,26]. Honeydew honey is a specific type of honey, obtained from the secretions of plants or aphids and, in some cases, insects’ excretions, especially when natural sources and climatic conditions favor the harvest of honeydew over nectar. Red and white clovers are preferred by several aphid species, such as *Aphis coronillae* Ferrari, *Therioaphis trifolii*, and *Acyrthosiphon pisum* Harris [27,28]. Honeydew honey is mainly studied for the variety of sugars it contains, such as α,α-trehalose, melezitose, theanderose, nystose, or maltotetraose in honeydew, as well as chemical indicators, enabling the differentiation of its types and the distinction from nectar honey [29,30]. Studies of honeydew collected from field beans infected with aphids (*Acyrthosiphon pisum*) were conducted in Belgium, revealing that the protein diversity of aphid honeydew originates from the host aphid and its microbiota, including endosymbiotic bacteria and gut flora [31].

This study aimed to determine monofloral red clover and rapeseed honey samples of Lithuanian origin at the protein level by describing and comparing their protein composition and aimed to evaluate proteins associated with aphids and lactic acid bacteria in honey samples.

## 2. Materials and Methods

### 2.1. Collection of Honey and Determination of Its Botanical Origin

Honey samples were collected in different districts of Lithuania. All honey samples were collected from Apis mellifera bees bred in Lithuania. Monofloral clover honey (C3) was collected from a private beekeeper farm in Rokiškis district, while monofloral rapeseed honey samples (S5 and S15) were collected in Kėdainiai, as well as polyfloral samples from Ukmergė (S22) and Rokiškis (S23) districts. Honey samples were tested after 6 months of storage in dark glass bottles in the refrigerator at 5 °C until used in further analysis. Honey sample preparation for botanical composition analysis was performed using the melissopalynology technique as described in [23]. In brief, a 10 g honey sample was weighed and dissolved in distilled water and centrifuged. The sediment was washed with 20 mL of distilled water and again centrifuged. Sediment was collected and spread on a slide over an area of approximately 20 mm × 20 mm, dried and covered with glycerine jelly. Pollen photos taken under a Nikon Eclipse E600 microscope (Nikon Corporation, Tokyo, Japan) at two positions: polar and equatorial view, at 400× magnification, focusing on pollen wall and surface sculpture. The botanical composition of honey was assessed by calculating the frequency of pollen in honey samples and expressed as a percentage of total pollen sum and considered as monofloral if the species was predominant and accounted for 45%; secondary pollen—16–45%; important pollen 3–15; minor pollen <3%. The botanical origin of the pollen was determined by taking photos and compared with the known plant pollen photos presented in the pollen catalogue [24,32].

### 2.2. Protein Isolation and Preparation for LC–MS

Proteins have been identified in the pollen separated from the honey. Pollen proteins were extracted as described in our previous study [33]. Briefly, pollens were homogenized in buffer, then lysed by boiling for 5 min at 95 °C and centrifuged for 30 min. The pellets containing proteins were precipitated using 5 vol of ice-cold 97.6% acetone, stored at −20 °C overnight, and afterwards the pellet was washed twice with 96.6% ethanol by centrifugation. The protein pellet was dissolved in 8 M urea solution and supplied for mass spectrometry analysis.

Whole proteome samples were digested with trypsin according to FASP protocol as described by Wiśniewski et al. [34]. Briefly, proteins were diluted in urea, alkylated and digested overnight with TPCK Trypsin 20233 (ThermoFisher Scientific, Vilnius, Lithuania), then centrifuged and additionally eluted using 20% CH_3_CN. The solution was acidified with 10% CF_3_COOH and lyophilized in a vacuum centrifuge. The lyophilized peptides were redissolved in 0.1% formic acid.

### 2.3. LC–MS^E^ (DIA)-Based Protein Identification

Liquid chromatography (LC) was performed using a Waters Acquity Ultra-Performance LC system (Waters Corporation, Wilmslow, UK) with an analytical column of ACQUITY UPLC HSS T3 250 mm. Data were acquired using the Synapt G2 mass spectrometer and Masslynx 4.1 software (Waters Corporation) in positive ion mode, using data-independent acquisition (DIA) coupled with ion mobility separation (IMS, *UDMS*^E^) [35]. For the survey scan, the mass range was set at 50–2000 Da with a scan time of 0.8 s. Raw data were lock mass-corrected using the doubly charged ion of [Glu1]-fibrinopeptide B (*m*/*z* 785.8426; [M+2H]^2+^) and a 0.25 Da tolerance window and processed with the ProteinLynx Global SERVER (PLGS) version 3.0.1 (Waters Corporation, Manchester, UK) Apex3D and Pep3D algorithms to generate precursor mass lists and associated product ion mass lists for subsequent protein identification and quantification. Peak lists were generated using the following parameters: (i) low energy threshold was set to 150 counts, (ii) elevated energy threshold was set to 50 counts, (iii) intensity threshold was set to 750 counts. Database searching was performed with the PLGS search engine using automatic peptide tolerance and fragment tolerance, minimum fragment ion matches of 1 per peptide and 3 per protein, and false discovery rate (FDR < 4%). Trypsin as the cleavage protease was used for data analysis, one missed cleavage was allowed, and the fixed modification was set to carbamidomethylation of cysteines, the variable modification was set to oxidation of methionine. UniProtKB/SwissProt databases were used for protein identification.

### 2.4. Statistical Analysis

Label-free quantification using the TOP3 approach was used for the quantification of proteins. TOP3 intensity was calculated as the average intensity of the three best ionizing peptides using ISOQuant [36]. The maximum FDR of protein identification was set to 1%. Identified proteins were submitted to *AgBase* (Version 2.0) (https://agbase.arizona.edu, accessed on 24 September 2021) for annotation of Gene Ontology (GO) functions.

## 3. Results

### 3.1. Comparison of the Protein Number of Monofloral Red Clover Honey with Other Honeys of Different Origins

**Figure 1:**
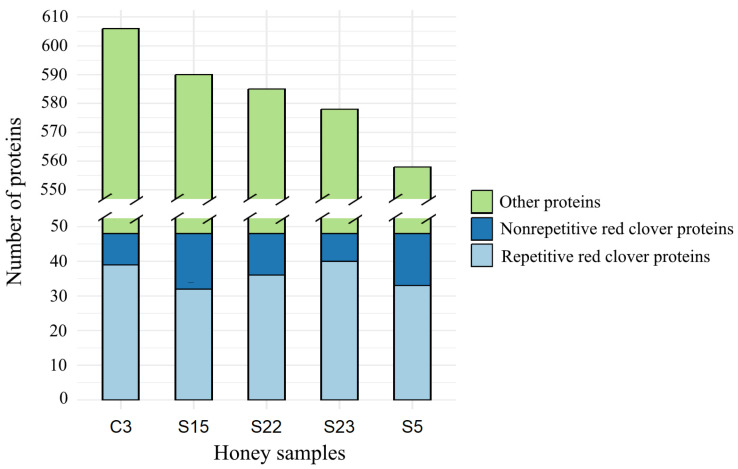
Number of proteins identified in five honey samples of Lithuanian origin, where C3: monofloral red clover honey; S5, S15, S22: monofloral rapeseed honey; S22: polyfloral honey.

**Figure 2:**
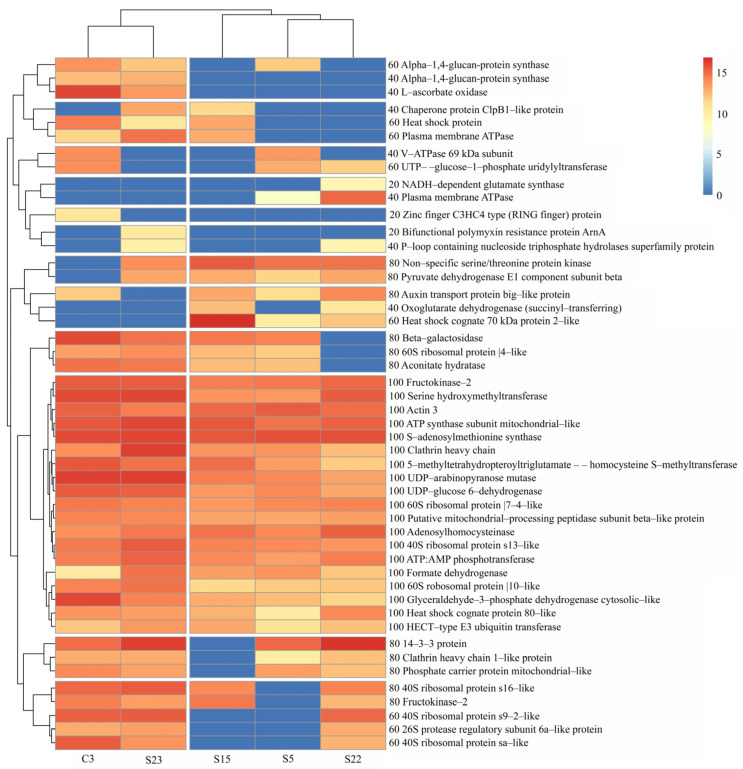
Heat map of the pattern of abundance of red clover protein (*Trifolium pratense*) (rows) in honey samples (columns). The color indicates the abundance of log2 proteins, and the blue indicates a missing value. The numbers before the names of the proteins indicate frequency of occurrence (%). Proteins common to all five honey samples are shown at different intensities of red color.

The total number of proteins identified in the studied honey samples ranged from 606 to 558, where C3 sample contained the highest number of proteins and S5 the lowest (Figure 1). The total number of identified red clover proteins was 240, where monofloral red clover (C3) and monofloral rapeseed (S23) honey samples showed the highest number of red clover proteins, 39 and 40, respectively, although no red clover pollen was found in sample S23. Though the majority of the identified red clover proteins were repetitive throughout all five honey samples, about 25% of red clover proteins were non-repetitive (Figure 1). A heat map was created for the comparison of the abundance of red clover proteins in different honey samples (Figure 2).

The highest frequency of occurrence was found for 19 red clover proteins in all honey samples studied. It was found that not every red clover protein was repeated in the individual honey tested. Proteins that are present only in honey samples S22, C3, and S23 are NADH-dependent glutamate synthase, plasma membrane ATPase, zinc finger C3HC4 type protein (RING finger) protein, and bifunctional polymyxin resistance protein ArnA, indicated by the yellow color in the heatmap. The relative composition of these non-repetitive proteins was the lowest compared to the other proteins present in the five honey samples studied, at 20.0%. Among six identified ribosomal proteins, three were identified in all samples, while a 40S s16-like ribosomal protein was present in all sample except S5. The other ribosomal proteins, 40S ribosomal protein s9-2-like (Fragment) and 40S ribosomal protein sa-like (Fragment), were not present in two honey samples, specifically S5 and S15, and the sequence coverage for the latter ribosomal proteins was low—7.41 and 12.50% (Table 1).

**Figure 3:**
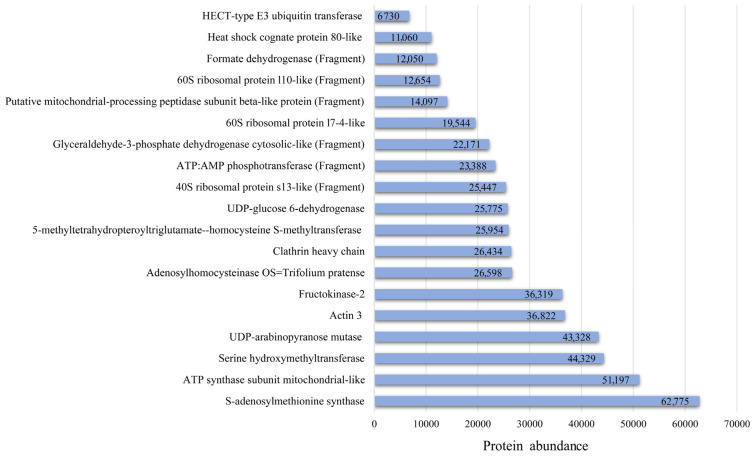
Composition and mean abundance of red clover proteins identified in tested honey samples.

The mitochondrial-like UDP-arabinopyranose mutase, actin 3, and ATP synthase subunit proteins had highest sequence coverage, 38.81%, 37.67%, and 33.33%, compared to all identified proteins in this study. A total of 19 red clover proteins were common to all tested honey samples of different botanical origin and comprised 39.6% of the total red clover proteins identified (Figure 3). The relative abundance of these 19 proteins ranged from 6730 to 62,775.1, with HECT-type E3 ubiquitin transferase being the lowest and S-adenosylmethionine synthase the highest.

The bifunctional polymyxin resistance protein ArnA was identified only in monofloral rapeseed honey (S23). NADH-dependent glutamate synthase was detected only in the honey sample S22.

### 3.2. Pollen Composition of Honey Samples from Different Regions of Lithuania

**Figure 4:**
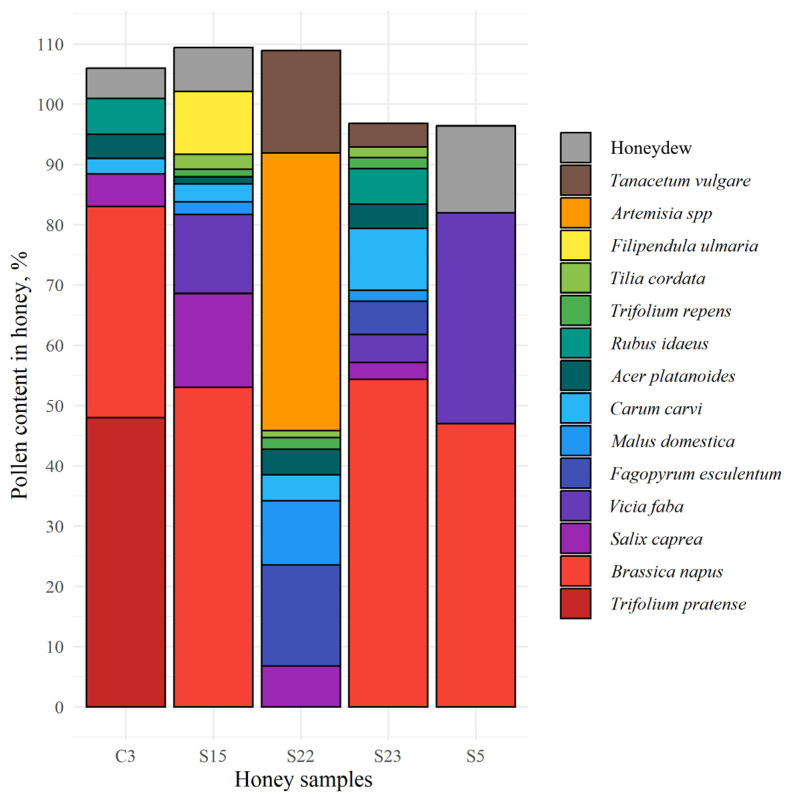
Botanical origin of five honey samples of Lithuanian origin, based on the pollen content, where C3: monofloral red clover honey; S5, S15, S22: monofloral rapeseed honey; S22: polyfloral honey.

The monofloral red clover honey (sample C3) contained 48% of red clover pollen, while rapeseed pollen comprised 35% of total pollen content (Figure 4). The amount of pollen in monofloral rapeseed honey samples S5, S15, and S23 ranged from 47.0 to 54.4% and polyfloral honey sample S22 contained 29.9% of pollen of this species. The secondary pollen of faba bean (*Vicia faba*) and thistle (*Cirsium vulgare*) was found in samples S5 and S22 comprising 35.0% and 21.1% of total pollen, respectively, while, in addition, sample S22 contained 16.8% of buckwheat (*Fagopyrum esculentum*) pollen. The important minor pollen, in particular willow (*Salix caprea*), meadowsweet (*Filipendula ulmaria*), and faba bean (*Vicia faba*), were found in the sample S15 and accounted for 15.6%, 13.1%, and 10.4%, respectively. The samples S22 and S23 contained other important minor pollen, such as fruit tree (*Malus domestica*) and caraway (*Carum carvi*), ranging from 10.6% to 10.3%, respectively. The lowest content of pollen from the latter group, namely, raspberry (*Rubus idaeus*), willow (*Salix caprea*), and maple (*Acer platanoides*), was found in sample C3 and accounted for 6.0%, 5.4%, and 4.0%, respectively. Abundant concentrations of anemophilous pollen (63.0%) were detected in polyfloral honey sample S22. Among them were sweet wormwood (*Artemisia annua*), accounting for 46.0%, and tansy (*Tanacetum vulgare*), accounting for 17.0%. The concentration of honeydew elements was higher in monofloral rapeseed honey samples S5 and S15, accounting for 14.4% and 7.3%, while only 5.0% of it was found in monofloral clover honey.

**Figure 5:**
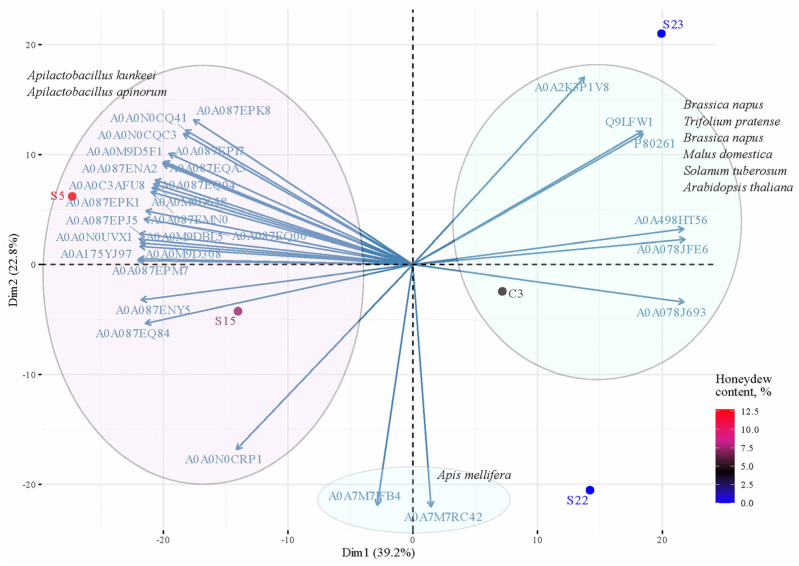
Principal component analysis based on the abundance of the top 30 proteins.

The principal component analysis (PCA) and the contribution of variables were calculated in R with the factoextra package (v.1.0.7) using transformed Log2 data of protein abundance (Figure 5). Top 30 proteins contributed to the principal components (total contribution of a given protein, on explaining the variations retained by two principal components). Proteins correlated with PC1 and PC2 are the most important in explaining variability in the data set.

### 3.3. Comparison of the Diversity of Plant Proteins Found in Honey Samples

Proteins related to 13 plants were detected in the honey samples (Table 2) and associated with pollen of 8 nectariferous and 5 anemophilous plants. Among them, there were mainly proteins associated with rapeseed (*Brassica napus*), the number of which varied within limits from 59 to 82 and consisted of 12.1%. Lower, but nevertheless relative, amounts of protein are associated with red clover (*Trifolium pratense*) and apple tree (*Malus domestica*) plants—6.2%, willow (*Salix viminalis*)—5.0%, and cherry (*Prunus avium*)—4.2%. The properties of the latter proteins have been reported in our previous studies [37,38,39].

There were only three proteins related to faba bean (*Vicia faba*), and their number was the same in all honey samples tested. Honey samples contain a large group of specific proteins for bees (*Apis mellifera*). The number of honeybee-specific proteins obtained in current study ranged from 59 to 61 and accounted for 10.3% of the total amount. Other plant proteins detected in this assay have been associated with pollen from anemophilous plants, e.g., arabidopsis (*Arabidopsis thaliana*), annual mugwort (*Artemisia annua*) and mugwort (*Artemisia keiskeana*)*,* carrot (*Daucus carota* subsp. Sativus), and potato (*Solanum tuberosum*), among which the *Artemisia annua* pollen was the highest at 7.4%.

### 3.4. The Identified Proteins of Aphids and Their Endosymbionts

Honeydew is a sweet and sticky liquid excreted by certain insects, usually aphids, and is collected by bees for honey production. The amount of honeydew found in the honey samples ranged from 2.4% to 14.4%. Detected aphid proteins associated with *Acyrthosiphon pisum* represent the largest group in comparison to *Aphis craccivora* and *Aphis glycines*, and varied within limits of 17–25, 10–16, and 4–11, respectively, in tested honey samples. Considering the relative amount of these protein numbers, we can say that they constitute small amounts of 3.5%, 2.2%, and 1.3% (Table 2). Only four single proteins related to the endosymbiont black bean aphid *Buchnera aphidicola* (*Aphis fabae*), *the* soybean aphid *Buchnera aphidicola* (*Aphis glycines*) and cotton aphid *Buchnera aphidicola* (*Aphis gossypii*) *were found* in the studied honey samples (Table 2). Facultative symbionts *Serratia symbiotica* is a species of bacteria endosymbiont of the black bean aphid *Aphis fabae* [30]. Slightly more (2–3) facultative endosymbionts proteins of *Serratia symbiotica* and the *Arsenophonus endosymbiont of Aphis craccivor were found in our honey samples* than those of *Buchnera aphidicola Aphis fabae.* The content of aphid endosymbionts in our honey samples was very low, between 0.07 and 0.5%.

### 3.5. Lactic Acid Bacteria in Honey

We identified proteins associated with *Apilactobacillus kunkeei* and *Apilactobacillus apinorum* in five honey samples studied; the number of proteins related to these bacteria prevailed in the range of 87–105 and 23–36, respectively (Table 3). The total numbers of these proteins present in all honey samples are 146 and 471, representing 16.1% and 5.0% of all proteins found in the samples. Proteins specific for bees (*Apis mellifera*) account for 10.3%, while other different LABs account for less than 1.0%. The total percentage of proteins associated with plants in honey samples is 58.84%. The same honey samples contained microbiota including aphis (*Acyrthosiphon pisum*), *Aphis craccivora*, and *Aphis glycines*, all LAB taken together, as well endosymbionts, and their numbers accounted for 41.07%. These data indicate that the number of plant proteins exceeds the microbiota present in the honey samples.

The data on protein content in honey show a slightly different trend compared to the differentiation in the protein number obtained from microbiota, plants, and bee-specific proteins. The following sequence of these data is obtained after evaluating the significant protein amounts: mean proteins content for *Apilactobacillus kunkeei* was 161.78 µg, plant proteins, *Apilactobacillus apinorum*, and *Apis mellifera* 130.78 µg, 48.56 µg, and 57.8 µg, respectively (Table 4). The relative amount for *Apilactobacillus kunkeei* is 40.55%, plant proteins 32.78%, while *Apilactobacillus apinorum* and *Apis mellifera* accounted for 21.17% and 14.49%, respectively.

Statistical analysis reveals significant differences between the protein content of rapeseed and all proteins related to the proteins of anemophilous plants at *p* < 0.05. The same trend was found between proteins associated with red clover and proteins characteristic of anemophilous plants, such as *Arabidopsis thaliana*, *Solanum tuberosum*, and *Daucus carota* subsp. *sativus*. However, there were no significant differences between the proteins associated with rapeseed oil and red clover proteins, as well as apple tree proteins.

Statistically significant differences in protein content are observed between *Apilactobacillus kunkeei*, *Apilactobacillus apinorum*, and *Apis mellifera*, at *p* < 0.05. Significant differences in protein content are also obtained between plant proteins compared to *Apilactobacillus apinorum* and *Apis mellifera* at *p* < 0.05, while differences were insignificant between the *Apilactobacillus kunkeei* and plant proteins groups. A significant linear relationship was determined between the content of *Apilactobacillus kunkeei* and *Apilactobacillus apinorum*, correlation coefficient (*r* = 0.97). Assuming that the identified proteins belong to aphids that live in leguminous plants, the relationship between the amount of faba bean (*Vicia faba*) pollen found in honey and the proteins associated with aphids and lactic acid bacteria was calculated (Table 2 and Table 4).

The strongest correlation coefficients were observed between FBP and LABN as well FBP and LABC, at *r* = 0.943 and 0.935, respectively. Moderate correlation (*r* = 0.764) was found between the LABC and LABN.

### 3.6. Gene Ontology (GO) Classification of Red Clover Proteins

#### 3.6.1. Evaluation of Red Clover Proteins According to Biological Processes

The red clover proteins were submitted to AgBase (version 2.0) and evaluated according to the Gene Ontology resource (GO), which consists of ternary parts [40]. The highest number of proteins related to red clover found in the tested honey samples was associated with the biological processes of metabolic process (eight), biosynthetic process (six), and translation (six). The other biological processes (four) include the metabolic process of cellular amino acids, the metabolic process of sulfur compounds, transport, and cofactor metabolic process (Tables S1 and S2). There is also a small-molecule metabolic process, glycolytic process, glycine biosynthetic process from serine, a cellular amino acid biosynthetic process, and five other processes. The biosynthetic process is associated with nucleoside monophosphate phosphorylation, methionine biosynthetic process nucleoside, S-adenosylmethionine biosynthetic process, and three other processes.

In total, five proteins were determined as involved in oxidation–reduction process (Tables S1 and S2), namely, phosphorylation—four; a carbon metabolic process—three; each of those processes included two proteins: (i) methylation; (ii) intracellular protein transport; (iii) clathrin coat assembly; a protein was in process of proton transmembrane transport process, and many other processes were also involved. The four proteins group involved in transport, sulfur compound metabolic process, cellular amino acid metabolic process, and cofactor metabolic process accounted for 9.5%. The three smallest protein groups involved in chromosome organization signal transduction and protein folding composed an equal part of 2.4%.

#### 3.6.2. Characteristics of Red Clover Proteins Annotated in the Biological Process and Results of Experimental Data

In this investigation, we present detailed protein characterization involved in the 125 different biological processes based on protein annotation. Proteins involved in oxidation–reduction included various proteins (Table S1). During the study of honey extracts, the following enzymes involved in the oxidation–reduction process were identified: L-ascorbate oxidase; pyruvate dehydrogenase E1 component subunit beta; oxoglutarate dehydrogenase (succinyl-transferring); NADH-dependent glutamate synthase (Fragment); and formate dehydrogenase (Fragment). The molecular weight and isoelectric point (IEP) of those enzymes were 62.1; 39.1; 117.2; 143.1; and 39.0 kDa and 8.91; 13.61; 1.96; 5.34; and 4.52, accordingly (Table 1).

The metabolic process (GO:00442810) included eleven different processes, and the proteins were identified in eight processes (Table S1). The enzyme identified in these processes, the beta subunit of the beta component of pyruvate dehydrogenase E1, is involved in the glycolytic process. Enzymes adenosylhomocysteinase, S-adenosylmethionine synthase, and serine hydroxymethyltransferase are involved in the metabolic process. The sequence coverage for proteins from a group of small molecule metabolic processes is higher compared to proteins involved in the oxidation–reduction process, and the coverage varies in the range of 5.34 to 22.98%. Our data reveal the process of tetrahydrofolate interconversion in the metabolic processes of the cellular nitrogen compound as well in small molecule metabolic processes. UTP--glucose-1-phosphate uridylyltransferase (Fragment) participates in the UDP-glucose metabolic process, and ATP:AMP phosphotransferase (Fragment) participates in the nucleoside monophosphate phosphorylation process. NADH-dependent glutamate synthase (Fragment) participates in the glutamate biosynthetic process. The isoelectric point of those enzymes varied from 5.67 to 7.17 and the molecular mass ranged from 42.0 to 143.1 kDa. The lowest molecular mass was determined for UTP--glucose-1-phosphate uridylyltransferase (Fragment) and highest for NADH-dependent glutamate synthase (Fragment) (Table 1). We establish the biosynthesis process that is included in the above-mentioned glycine biosynthetic process mentioned above from serine (A0A2K3NMF1), cellular amino acid biosynthetic process (A0A2K3P3K7), and others, such as methionine biosynthetic process (A0A2K3P3K7), glutamate biosynthetic process (A0A2K3PCD9), nucleoside monophosphate phosphorylation (A0A2K3MUW7), and acetyl-CoA biosynthetic process from pyruvate (A0A2K3NQ11).

The protein translation process is related to ribosomal proteins. Data reveal six ribosomal proteins: 40S ribosomal protein s9-2-like (Fragment), 40S ribosomal protein s16-like, 40S ribosomal protein s13-like (Fragment), 60S ribosomal protein l10-like (Fragment), 40S ribosomal protein sa-like (Fragment), and 60S ribosomal protein l4-like (Fragment). The data show that these proteins are characterized by high isoelectric points 10.63–11.09, except 40S ribosomal protein sa-like (Fragment), which was extracted at isoelectric point of 5.07. This indicates that the latter proteins are exclusively basic. The molecular weight of the identified ribosomal proteins ranged from 13.6 to 28.6 kDa and the number of reported peptides ranged from 2 to 6. The sequence coverage (31.09%) was highest for the 40S ribosomal protein s13-like (Fragment); lowest (7.41%) for the 40S ribosomal protein s9-2-like (Fragment), and for others from 17.86% to 21.05%.

#### 3.6.3. Evaluation of Red Clover Proteins According to Molecular Functions

The molecular function usually is a single-step reaction. We present red clover proteins according to the annotations determined for 128 molecular functions that were detected from experimental study of honey samples (Table 1 and Table S1). Among the proteins whose molecular functions were determined are the activity of phosphotransferase and an alcohol group as acceptor (Table S1). This protein catalyzes the transfer of a phosphorus-containing group from a compound (donor) to an alcohol group (acceptor).

The other proteins with molecular function annotated in the GO description and related to red clover proteins involve two different areas (Tables S1 and S3). Data corresponding to molecular functions of proteins include protein serine/threonine kinase activity; hydrolase activity; methionine adenosyltransferase activity; adenosylhomocysteinase activity; adenylate kinase activity; transferase activity; catalytic activity; peptidase activity; protein serine/threonine kinase activity; kinase activity; adenylate kinase activity; and others. The molecular function is associated with proteins and the metal ions binding process. Our data reveal a molecular binding function for those proteins: NAD binding; pyridoxal phosphate binding; nucleotide binding; coenzyme binding; thiamine pyrophosphate binding; ADP binding; protein domain-specific binding; clathrin light chain binding; ATP binding; RNA binding; and rRNA binding, as well as others (Table S1). In the studied honey samples, we identified zinc ion binding; copper ion binding; and metal ion binding as well as some clusters with iron: 4 iron, 4 sulfur cluster binding; and iron–sulfur cluster binding. The study determined the number of proteins involved in various molecular functions (Table S1).

#### 3.6.4. Evaluation of Red Clover Proteins According to the Cellular Components or Macromolecular Complexes

Gene Ontology analysis of the cellular components reveals 78 terms of the cell components based on the data of the annotation results (Table S1). The major cell components include the nucleus; nucleoplasm; cytoplasm; and Golgi apparatus. According to the GO analysis, the cellular components of red clover include small ribosomal subunit; mitochondrial matrix; clathrin complex; proton-transporting V-type ATPase, V1 domain; clathrin coat of the trans-Golgi network vesicle; and clathrin coat of the coated pit (Table S1). Various membranes were identified, including cytoplasmic vesicle membrane; integral component of the membrane; and plasma membrane (Tables S1 and S4). Protein-containing complexes were found as assemblies that contains small ribosomal subunit; clathrin complex; clathrin coat of trans-Golgi network vesicle; clathrin coat of coated pit and histone acetyltransferase complex; and proton-transporting V-type ATPase, V1 domain (Tables S1 and S4).

## 4. Discussion

The protein composition of five honey samples was investigated, among which one sample was monofloral red clover honey. However, all samples of honey contained proteins related to red clover proteins. Seventeen red clover proteins whose peptides were sequenced had protein coverage greater than 20.0%, and five proteins ranged from 17.86% to 10.34%. A total of 26 proteins out of 48 studied had a sequence coverage of 9.97 to 0.59%, among which the lowest was HECT-type E3 ubiquitin transferase (Table 1). A total of 331 functions of red clover proteins were annotated, among which 125 are involved in biological processes, 128 have a molecular function, and 78 are related to the components of cell structure (Table S1). The molecular function of the proteins studied was associated with binding, catalytic, transporter, structural, and nucleic acid binding transcription factor activities, as well as with components of membranes, organelle, and protein-containing complexes. The red-clover-related proteins have been identified in various cellular components are as follows: histone acetyltransferase complex, proton-transporting V-type ATPase, V1 domain, and clathrin complex (Table S1).

The protein sequence-based prediction method was applied for the annotation of protein function of leguminous plant species such as barrel clover (*Medicago truncatula*) and soybean (*Glycine max*), as well as other plants among which were rice (*Oryza sativa*), poplar (*Populus trichocarpa*), and tomato (*Solanum lycopersicum*) [41]. GO analysis of the above-mentioned and the additional five species of *Trifolium subterraneum*, *Medicago truncatula*, *Cicer arietinum*, *Trifolium pratense*, and *Glycine max* confirms the genes involved in biological processes assist in metabolic, cellular, single-organism, localization, biological regulation, and signal processes, etc., and this has been reported in earlier studies [42].

The two proteins identified in honey samples extract are V-ATPase 69 kDa subunit and clathrin heavy chain (Table 1). We have identified L-ascorbate oxidase belonging to the family of multi-copper oxidases and function in plants and fungi [43] by oxidizing ascorbic acid to dehydro-L-ascorbic acid, a potentially toxic product damaging the digestive system of the herbivore *Helicoverpa zea*. Oxidized ascorbate loses its antioxidant properties, as well as its nutritional and antioxidant functions in phytophagous insects and, thus, has a potentially important role as a plant defense protein against insects [44]. The other enzyme of the oxidase family, namely, glucose oxidase (GOD), with a molecular weight (Mw) of approximately 170 kDa, was identified in buckwheat honey [45]. In this origin of honey, GOD had the highest enzymatic activity of 1.13 µmol min^−1^/g^−1^, compared to rapeseed (0.55 µmol min^−1^/g^−1^) or willow honey (0.1–0.51 µmol min^−1^/g^−1^) [46].

Auxin is a plant hormone playing an important role in plant growth and signaling, and it regulates photosynthetic rates and chlorophyll content in many plant species [47]. The BIG auxin transport protein is involved in auxin efflux and polar auxin transport (PAT) [48]. Auxin transport big-like protein, with a molecular weight of 573.9 kDa and isoelectric point pI of 5.78, was found in four of the honey samples studied, among which was monofloral clover honey (Table 1). It was annotated as the protein is involved in the binding of zinc ions (Table S1). It has a high molecular weight of 498.9 kDa and was annotated as zinc finger protein (ring finger) protein (Fragment), and was identified in monofloral clover honey. The study by Mohanta and colleagues [49] on the plant proteome reveals that higher eukaryotic plants contain five proteins with higher molecular mass compared to others, among which is auxin transport protein BIG 568.4 kDa; the auxin protein (auxin TP BI) was identified in the molecular weight of *Trifolium pratense* of 566.6 kDa compared to other proteins in this plant. The study reveals that proteins with acidic pI predominate over the proteins with an alkaline pI, suggesting that it depends on differential composition of amino acids in different species, and this might be associated with environmental and ecological pressure. The enzyme S-adenosylmethionine synthase, also known as methionine adenosyltransferase (MAT), was determined during our analysis of honey samples. According to research data, this enzyme catalyzes the formation of S-adenosylmethionine (AdoMet, SAM, or SAMe) from methionine and ATP [50,51]. The SAM component is a precursor for the plant hormone ethylene [52].

UTP-glucose-1-phosphate uridylyltransferase, also known as glucose-1-phosphate uridylyltransferase (UDP–glucose pyrophosphorylase) or UGPase, is an enzyme involved in carbohydrate metabolism and has been used to improve the quality and increase the production of agricultural plants [53,54,55]. This enzyme was identified in the honey samples we studied, supporting the earlier findings of Treigytė and coauthors [11], who found protein uridine diphosphate (UDP)-glucose 6-dehydrogenase 4 and UDP-glucose 6-dehydrogenase 5 with a molecular weight of 56 kDa and 55 kDa, respectively, in red clover pollen. Glucose-1-phosphate uridylyltransferase catalyzes the formation of uridine diphosphate-glucose (UDP-glucose) from glucose-1-phosphate and uridine-5′-triphosphate (UTP) [56].

The sucrose–phosphate synthase enzyme catalyzes the transfer of a uridine diphosphate glucose (UDP-glucose) to D-fructose 6-phosphate to form UDP and D-sucrose-6-phosphate, and in the following step, hydrolyzes D-sucrose-6-phosphate to sucrose [57,58]. Sucrose phosphate synthase is an essential enzyme in sucrose synthesis, suggesting that the expression of UTP-glucose-1-phosphate uridylyltransferase in plant nectar affects the formation of UDP-glucose, which is an essential component in carbohydrate metabolism. Uridine diphosphate glucose (UDP-glucose) is a nucleotide sugar involved in the synthesis of cellulose, hemicellulose, and pectins for the production of plant cell walls [59]. Nucleotide sugars such as UDP-glucose, GDP-fucose, UDP-xylose, and UDP-N-acetyl galactosamine are transported from one UDP-sugar to another for the exchange of various nucleotides [60]. The transport of UDP-sugars in cells takes place through specific membrane-bound protein transporters. The synthesized compounds are used for the production of glycoproteins and glycolipids [61]. Nitrogen fixation chloroplastic NifU-like protein 3, known for its functions in protecting plants against abiotic and biotic stresses [62], was identified in spring honey collected by bees mainly from orchards or manually collected directly from orchard blooms [37], showing its higher expression in manually collected pollen of apple tree (*Malus sylvestris*) and plum (*Prunus*) compared to other orchard tree pollen. The concentration range of the bee-collected pollen of the *Prunus* and willow (*Salix* spp.) mixture was close to that of monofloral *Prunus* pollen. Purified NifU is a red protein that contains iron in the form of a redox-active [2Fe-2S]^2+,+^ cluster. The primary structure consists of the three conserved cysteine residues that are involved in the assembly of a transient FeS cluster [63]. It was stated that transient [2Fe-2S]^2+,+^ cluster units are formed on NifU and subsequently released to provide the inorganic iron and sulfur required for the maturation of the nitrogenase component proteins [64]. NifU-type proteins forming iron–sulfur clusters can also be found in organisms that do not fix nitrogen [65]. Although we did not observe NifU-like protein in the honey samples studied, other enzymes with iron–sulfur clusters, in particular iron–sulfur protein aconitate hydratase containing [4Fe-4S] cluster, was determined.

Aconitate hydratase, later named aconitase, belongs to the aconitase/isopropylmalate (IPM) isomerase family, comprised of three classes of hydrolyase enzymes: aconitases, homoaconitases, and IPM isomerases. These enzymes have the same Fe-S cluster-binding site in their structure [66]. According to research data, tricarboxylic acid cycle (TCA), also known as the Kreb’s/Citric Acid cycle, is activated in bacteroids during nitrogen fixation. The components of TCA are the main carbon source for bacteroids [67]. The aconitase (AcnA), as well as other seven proteins from TCA, were identified by the proteomics analysis in nitrogen fixation bacteroid *Rhizobium Etli*. These data were obtained during the experiment when the root nodules of the bean plant (*Phaseolus vulgaris*) were infected by *R. etli* (*Phaseolus vulgaris*) [68].

Aphids, such as the genera *Cinara* and *Physokermes*, which have spread in forests in coniferous trees, including spruce and fir, feed on the sap of the phloem of these plants and produce honeydew honey with a varying composition of sugar, amino acids, and several inorganic ions, among which potassium ions (K+) and phosphate (PO4^3−^) are the most abundant anion in honeydew honey [69]. The pea aphid, *Acyrthosiphon pisum* (Harris), infests and can cause huge damage to leguminous plants, such as faba bean (*Vicia faba* L.), lupin (*Lupinus albus* L.), alfalfa (*Medicago sativa* L.), lentil (*Lens culinaris* Medik.), chickpea (*Cicer arietinum* L.), grass pea (*Lathyrus sativus* L.), pea (*Pisum sativum* L.), and clover (*Trifolium subterraneum*) [70,71]. *Acyrthosiphon pisum* was reported to prefer faba bean and clover [72] and can transmit phytoviruses and cause more than 20 species of plant virus diseases [73,74]. Though red clover aphids (*Aphis coronilla* Ferrari (Hemiptera: Aphididae)) are widely distributed in Europe in pulses [75], mostly on red clover and lucern, we did not identify any of their proteins in the studied honey, suggesting that these pests are not widespread in the Lithuanian legume fields.

The evolution of symbionts along with their hosts existed million years ago as a long-term mutualism. Symbionts are divided into obligate or primary and other facultative or secondary, as well-named, S-symbionts [76]. The primary endosymbiont of pea aphid, *Buchnera aphidicola*, synthesizes essential amino acids for its aphid host. About 9% of the *Buchnera* genome produces essential amino acids for the aphid [77]. Some new members of the *Lactobacillus* genus currently belong to the genus *Apilactobacillus*, which includes different species. *Apilactobacillus kunkeei* (basonym *Lactobacillus kunkeei*) and *Apilactobacillus apinorum* (basonym *Lactobacillus apinorum*) are incorporated into the genus *Apilactobacillus*. These LABs are associated with honeybees (*Apis mellifera*) and flowers. Lactic acid bacteria (LAB) are common inhabitants of the honeybee intestinal tract and are found in fresh honey [78,79]. The authors report that the bee gastric microbiota is dominated by lactobacilli and bifidobacteria. These bacteria are also found in flowers, nectar, fruits, and fermented foods. We identified *Lactiplantibacillus plantarum*, *Lactiplantibacillus acidophilus*, *Lactiplantibacillus amylovorus*, and *Lactiplantibacillus delbrueckii* subsp. *bulgaricus*. Most of these proteins were single or absent in the honey samples tested. The strains *L. delbrueckii* were mainly isolated from dairy products, including cheeses, yogurts, and fermented milk. The data reveal nine species of *L. delbrueckii*, which are probiotics. The probiotic strains of these bacteria have genes involved in various metabolic processes that affect the organoleptic properties of dairy [80]. In the honey we studied, these bacteria were identified in four samples out of five examined, and their number was very low (1–2); 2–4 proteins were found from *Lactiplantibacillus plantarum* and 2–3 proteins from *Lactiplantibacillus acidophilus*. Fructophilic LABs have been found in the digestive tracts of pollinators such as bees, bumblebees, and insects that consume significant amounts of fructose [81]. The prevalence of *Apilactobacillus kunkeei*, *Lactiplantibacillus plantarum*, and *Fructobacillus fructosus* was found in beebread, while low interspecies biodiversity of LAB *Apilactobacillus kunkeei* was found in the midgut of *Apis mellifera ligustica* honeybee [82]. *A. kunkeei* belongs to fructophilic (FLAB), a lactic acid bacteria of a subgroup of LAB, and grows on fructose, unlike other LAB, that grow on glucose [83]; thus, it often is found in environments associated with bees as high-fructose-consuming insects, on flowers, fruits, and also in fermented food that is produced from fruits [84,85]. The fructophilic strain of *L. plantarum* was isolated from honeydew honey collected in Poland [81] and since Poland and Lithuania are neighboring countries with the same climatic conditions, it suggests that the honeydew composition may be similar in terms of fructophilic lactic acid bacteria.

## 5. Conclusions

1. Studies of the botanical composition of honey pollen and the identification of honey extracted plant proteins of pollen have provided new data on the composition of proteins. Red clover pollen was found only in monofloral red clover honey, while red-clover-related proteins were found in all honey samples. It can be assumed that bees can reach red clover nectar not only from their own nectarines, but also from the bottom of the flowers.
2. Individual honey samples contained repetitive proteins common to all samples studied. Our data show that more repetitive red clover proteins were identified in each honey sample compared to some proteins that were not repeated in honey samples. The common number of proteins found in all honey samples was 39.6% of the total number of proteins found in each honey sample.
3. Data from the annotation results show that the most predominant molecular functions of red clover proteins in honey samples were related to ion binding to ATP, while others are metal ion binding; zinc ion binding; copper ion binding; pyridoxal phosphate binding; and thiamine pyrophosphate binding.
4. Analysis of the gene ontology of cellular components revealed single cell elements, various membranes, and macromolecular compounds. Some protein complexes that have been identified in the cell composition are histone acetyltransferase complex, proton-transporting V-type ATPase, V1 domain, and clathrin complex.
5. Proteins associated with aphids, such as pea aphid, as well as endosymbiont proteins, were identified in honey, among which the largest number of proteins was from *Acyrthosiphon pisum*.
6. In the honey samples tested, the lactic acid bacteria *Lactobacillus kunkeei* was found in higher concentrations than *Apilactobacillus apinorum*. *Lactiplantibacillus plantarum* and *Lactiplantibacillus acidophilus* were found in small amounts and *Lactiplantibacillus acidophilus*, *Lactiplantibacillus delbrueckii subsp. Bulgaricus* was solitary or unidentified in some samples.

## Figures and Tables

**Table 1:** Characterization of red clover (*Trifolium pratense*) proteins extracted from monofloral red clover and rapeseed honey, as well as multifloral honey.

Accession Number	Entry	Description	Mw (Da)	pI (pH)	NRP ^1^	SC ^2^, (%)
A0A2 K3P3K7	A0A2K3P3K7_TRIPR	5-methyltetrahydropteroyltriglutamate-homocysteine S-methyltransferase OS = T. pratense OX = 57577 GN = L195_g006435 PE = 3 SV = 1 *	84,808	6.21	15	22.35
A0A2K3N8B1	A0A2K3N8B1_TRIPR	Actin 3 OS = Trifolium pratense OX = 57577 GN = actin 3 PE = 3 SV = 1 *	42,047.0	5.08	13	37.67
A0A2K3PQG8	A0A2K3PQG8_TRIPR	14-3-3 protein OS = T. pratense OX = 57577 GN = L195_g014284 PE = 3 SV = 1	29,428	4.47	6	28.85
A0A2K3PKL4	A0A2K3PKL4_TRIPR	UDP-arabinopyranose mutase OS = T. pratense OX = 57577 GN = L195_g012535 PE = 3 SV = 1 *	40,749.8	5.75	10	38.81
A0A2K3NAQ8	A0A2K3NAQ8_TRIPR	ATP synthase subunit mitochondrial-like OS = Trifolium pratense OX = 57577 GN = L195_g023402 PE = 3 SV = 1 *	36,945.3	8.68	9	33.33
A0A2K3P1V8	A0A2K3P1V8_TRIPR	S-adenosylmethionine synthase OS = T. pratense OX = 57577 GN = L195_g005826 PE = 3 SV = 1 *	43,170.9	5.95	9	21.03
A0A2K3N8U2	A0A2K3N8U2_TRIPR	Glyceraldehyde-3-phosphate dehydrogenase cytosolic-like (Fragment) OS = Trifolium pratense OX = 57577 GN = L195_g022684 PE = 3 SV = 1 *	27,983.8	6.12	8	34.23
A0A2K3MUC7	A0A2K3MUC7_TRIPR	Adenosylhomocysteinase OS = T. pratense OX = 57577 GN = L195_g017604 PE = 3 SV = 1 *	53,696.5	5.90	12	27.01
A0A2K3M758	A0A2K3M758_TRIPR	Alpha-1,4-glucan-protein synthase OS = Trifolium pratense OX = 57577 GN = L195_g042687 PE = 4 SV = 1	24,287.1	6.33	4	26.67
A0A2K3M1W9	AOA2K3M1W9_TRIPR	40S ribosomal protein s9-2-like (Fragment) OS = T. pratense OX = 57577 GN = L195_g040850 PE = 3 SV = 1	16,395.1	10.72	2	7.41
A0A2K3PJ70	A0A2K3PJ70_TRIPR	Plasma membrane ATPase OS = Trifolium pratense OX = 57577 GN = L195_g012022 PE = 3 SV = 1	89,990.8	4.85	7	9.5
A0A2K3NU12	A0A2K3NU12_TRIPR	Aconitate hydratase OS = T. pratense OX = 57577 GN = L195_g002996 PE = 3 SV = 1	107,854.8	7.93	11	14.80
A0A2K3NL25	A0A2K3NL25_TRIPR	L-ascorbate oxidase OS = T. pratense OX = 57577 GN = L195_g000155 PE = 3 SV = 1	62,128.5	8.93	4	8.91
A0A2K3P8U8	A0A2K3P8U8_TRIPR	Plasma membrane ATPase OS = T. pratense OX = 57577 GN = L195_g008323 PE = 3 SV = 1	105,769.6	6.27	6	8.66
A0A2K3L1T3	A0A2K3L1T3_TRIPR	UDP-glucose 6-dehydrogenase OS = Trifolium pratense OX = 57577 GN = L195_g028356 PE = 3 SV = 1 *	54,262.1	6.34	10	21.56
A0A2K3NLX6	A0A2K3NLX6_TRIPR	V-ATPase 69 kDa subunit OS = T. pratense OX = 57577 GN = L195_g000444 PE = 3 SV = 1	68,962.7	5.08	14	32.26
A0A2K3LNL7	A0A2K3LNL7_TRIPR	Heat shock protein OS = T. pratense OX = 57577 GN = L195_g036127 PE = 3 SV = 1	59,084.3	5.13	4	7.41
A0A2K3NRS2	A0A2K3NRS2_TRIPR	Fructokinase-2 OS = T.pratense OX = 57577 GN = L195_g002190 PE = 4 SV = 1 *	31,966.5	5.66	6	21.69
A0A2K3PGB1	A0A2K3PGB1_TRIPR	Beta-galactosidase OS = T. pratense OX = 57577 GN = L195_g011002 PE = 3 SV = 1	87,315.5	8.55	5	6.33
A0A2K3NMF1	A0A2K3NMF1_TRIPR	Serine hydroxymethyltransferase OS = T. pratense OX = 57577 GN = L195_g000637 PE = 3 SV = 1 *	52,275.3	6.65	6	20.81
A0A2K3PJL4	A0A2K3PJL4_TRIPR	Fructokinase-2 OS = T. pratense OX = 57577 GN = L195_g006176 PE = 4 SV = 1	38,560.9	4.69	3	14.08
A0A2K3LKQ2	A0A2K3LKQ2_TRIPR	40S ribosomal protein s13-like (Fragment) OS = T. pratense OX = 57577 GN = L195_g035087 PE = 3 SV = 1 *	13,628.1	10.63	4	31.09
A0A2K3MF29	A0A2K3MF29_TRIPR	40S ribosomal protein s16-like OS = T. pratense OX = 57577 GN = L195_g041963 PE = 3 SV = 1	16,297.1	10.68	3	17.86
A0A2K3NCQ3	A0A2K3NCQ3_TRIPR	Heat shock cognate 70 kDa protein 2-like (Fragment) OS = T. pratense OX = 57577 GN = L195_g024109 PE = 4 SV = 1	97,918.3	5.60	2	3.46
A0A2K3NWT6	A0A2K3NWT6_TRIPR	UTP--glucose-1-phosphate uridylyltransferase (Fragment) OS = T. pratense OX = 57577 GN = L195_g004002 PE = 3 SV = 1	41,977.1	5.67	8	22.98
A0A2K3PGD6	A0A2K3PGD6_TRIPR	Alpha-1,4-glucan-protein synthase OS = Trifolium pratense OX = 57577 GN = L195_g011040 PE = 3 SV = 1	38,857.7	5.62	2	7.49
A0A2K3JM62	A0A2K3JM62_TRIPR	40S ribosomal protein sa-like (Fragment) OS = T. pratense OX = 57577 GN = L195_g048757 PE = 3 SV = 1	24,945.21	5.07	2	12.50
A0A2K3NK64	A0A2K3NK64_TRIPR	Putative mitochondrial-processing peptidase subunit beta-like protein (Fragment) OS = T. pratense OX = 57577 GN = L195_g026750 PE = 4 SV = 1 *	29,192.50	7.31	3	10.34
A0A2K3PKP0	A0A2K3PKP0_TRIPR	60S ribosomal protein l7-4-like OS = T. pratense OX = 57577 GN = L195_g012568 PE = 3 SV = 1 *	28,587.59	10.40	6	20.90
A0A2K3MW20	A0A2K3MW20_TRIPR	26S protease regulatory subunit 6a-like protein OS = T. pratense OX = 57577 GN = L195_g018159 PE = 3 SV = 1	42,632.90	5.26	2	8.44
A0A2K3LNN3	A0A2K3LNN3_TRIPR	60S ribosomal protein l10-like (Fragment) OS = T. pratense OX = 57577 GN = L195_g036135 PE = 3 SV = 1 *	19,939.28	10.91	4	21.05
A0A2K3PN04	A0A2K3PN04_TRIPR	Heat shock cognate protein 80-like OS = T. pratense OX = 57577 GN = L195_g013385 PE = 3 SV = 1 *	80,416.30	4.74	4	6.44
A0A2K3NMQ3	A0A2K3NMQ3_TRIPR	Clathrin heavy chain OS = T. pratense OX = 57577 GN = L195_g000733 PE = 3 SV = 1 *	194,249.43	5.12	5	3.93
A0A2K3NL88	A0A2K3NL88_TRIPR	Zinc finger C3HC4 type (RING finger) protein (Fragment) OS = T. pratense OX = 57577 GN = L195_g000168 PE = 4 SV = 1	498,961.65	5.45	4	1.59
A0A2K3MUW7	A0A2K3MUW7_TRIPR	ATP:AMP phosphotransferase (Fragment) OS = T. pratense OX = 57577 GN = L195_g017796 PE = 3 SV = 1 *	23,286.85	7.17	2	9.52
A0A2K3PPZ5	A0A2K3PPZ5_TRIPR	Formate dehydrogenase (Fragment) OS = T. pratense OX = 57577 GN = L195_g012685 PE = 3 SV = 1 *	39,037.45	6.12	2	4.52
A0A2K3NV46	A0A2K3NV46_TRIPR	Phosphate carrier protein mitochondrial-like (Fragment) OS = T. pratense OX = 57577 GN = L195_g003373 PE = 3 SV = 1	28,095.87	9.88	2	7.66
A0A2K3P5Y0	A0A2K3P5Y0_TRIPR	Oxoglutarate dehydrogenase (succinyl-transferring) OS = T. pratense OX = 57577 GN = L195_g007276 PE = 3 SV = 1	117,275.66	6.61	2	1.96
A0A2K3NKS8	A0A2K3NKS8_TRIPR	Non-specific serine/threonine protein kinase OS = T. pratense OX = 57577 GN = L195_g000037 PE = 4 SV = 1	451,716.00	6.54	2	1.67
A0A2K3MA51	A0A2K3MA51_TRIPR	Chaperone protein ClpB1-like protein (Fragment) OS = T. pratense OX = 57577 GN = L195_g043754 PE = 4 SV = 1	49,117.09	5.40	2	6.76
A0A2K3PPN3	A0A2K3PPN3_TRIPR	Bifunctional polymyxin resistance protein ArnA OS = T. pratense OX = 57577 GN = L195_g013962 PE = 3 SV = 1	43,325.58	6.73	2	6.82
A0A2K3NQ11	A0A2K3NQ11_TRIPR	Pyruvate dehydrogenase E1 component subunit beta OS = T. pratense OX = 57577 GN = L195_g001564 PE = 4 SV = 1	39,140.65	5.61	4	13.61
A0A2K3NL22	A0A2K3NL22_TRIPR	Auxin transport protein big-like protein OS = T. pratense OX = 57577 GN = L195_g000147 PE = 3 SV = 1	573,903.34	5.78	6	1.30
A0A2K3NLG1	A0A2K3NLG1_TRIPR	HECT-type E3 ubiquitin transferase OS = T.pratense OX = 57577 GN = L195_g000287 PE = 4 SV = 1 *	391,925.50	4.91	2	0.59
A0A2K3LUX0	A0A2K3LUX0_TRIPR	60S ribosomal protein l4-like (Fragment) OS = T. pratense OX = 57577 GN = L195_g038328 PE = 3 SV = 1	40,718.52	11,09	3	8.40
A0A2K3P6Y1	A0A2K3P6Y1_TRIPR	Clathrin heavy chain 1-like protein (Fragment) OS = T. pratense OX = 57577 GN = L195_g007628 PE = 4 SV = 1	140,432.72	4.94	5	6.23
A0A2K3N3T6	A0A2K3N3T6_TRIPR	P-loop containing nucleoside triphosphate hydrolases superfamily protein (Fragment) OS = T.pratense OX = 57577 GN = L195_g020931 PE = 3 SV = 1	69,425.16	5.17	4	9.97
A0A2K3PCD9	A0A2K3PCD9_TRIPR	NADH-dependent glutamate synthase (Fragment) OS = T. pratense OX = 57577 GN = L195_g009558 PE = 3 SV = 1	143,094.74	5.94	3	5.34

Notes: ^1^—number of reported peptides for protein (NRP); ^2^—sequence coverage (SC); *—proteins.

**Table 2:** Number of proteins associated with different floral and microbiota origins found in honey samples.

Plant, Microbiota, and Bee-Specific Proteins, Determined in Honey	Honey Samples					Total Number	Number of Proteins Expressed in %
	C3	S5	S15	S22	S23		
	Number of Proteins in Honey Samples						
Proteins associated with the pollen of nectariferous^N^ and anemophilous^AN^ plants							
*Brassica napus* ^N^	82	59	69	67	77	354	12.1
*Trifolium pratense* ^N^	39	33	32	36	40	180	6.2
*Malus domestica* ^N^	37	34	29	47	34	181	6.2
*Salix viminalis* ^N^	31	23	28	28	37	147	5.0
*Prunus avium* ^N^	24	22	22	29	26	123	4.2
*Vicia faba* ^N^	3	3	3	3	3	15	0.5
*Cirsium eriophorum* ^N^	1	0	0	0	0	1	0.3
*Vicia ramuliflora* ^N^	1	1	1	2	0	5	0.2
*Artemisia annua* ^AN^	45	33	47	50	42	217	7.4
*Daucus carota subsp. sativus* ^AN^	36	35	35	37	33	176	6.0
*Solanum tuberosum* ^AN^	34	28	35	32	35	164	5.6
*Arabidopsis thaliana* ^AN^	35	26	30	31	30	152	5.2
*Artemisia keiskeana* ^AN^	1	1	1	1	1	5	0.2
Proteins associated with *Apis mellifera*							
*Apis mellifera*	60	59	61	60	60	300	10.3
Proteins associated with aphids^A^, endosiombionts of aphis^E^, lactic acid bacteria^L^							
*Acyrthosiphon pisum* ^A^	22	19	25	17	19	102	3.5
*Aphis craccivora* ^A^	11	12	16	14	10	63	2.2
*Aphis glycines* ^A^	9	11	8	6	4	38	1.3
*Buchnera aphidicola (Aphis fabae*)^E^	0	1	1	0	0	2	0.07
*Buchnera aphidicola (Aphis glycines*)^E^	0	1	0	0	0	1	0.3
*Buchnera aphidicola (Aphis gossypii*)^E^	0	1	0	0	0	1	0.3
*Serratia symbiotica* ^E^	3	3	3	2	3	14	0.5
*Arsenophonus endosymbiont of aphis craccivor* ^E^	3	3	3	2	2	13	0.5
*Apilactobacillus kunkeei* ^L^	92	105	98	89	87	471	16.1
*Apilactobacillus apinorum* ^L^	27	36	34	23	26	146	5.0
*Lactiplantibacillus amylovorus* ^L^	0	1	0	0	1	2	0.7
*Lactiplantibacillus plantarum* ^L^	4	2	3	3	2	14	0.5
*Lactiplantibacillus acidophilus* ^L^	3	2	2	2	3	12	0.4
*Lactiplantibacillus delbrueckii* subsp. *bulgaricus*^L^	1	2	1	0	1	5	0.2
Proteins associated with bacteria^B^ and viruses^V^, animal-related^AR^							
*Escherichia coli* (*strain K12*)^B^	1	1	1	1	1	5	0.2
*Fagopyrum esculentum endornavirus 1* ^V^	0	0	1	1	0	2	0.7
Grand total	606	558	590	585	578	2917	100.0

**Table 3:** The significant protein content (µg) in honey samples of different floral origins.

Uniprot Accession Number	Protein Name	Species	C3	S5	S15	S22	S23
A0A078J693	BnaC03g73810D protein	*Brassica napus*	64.0	25.4	12.0	31.9	47.6
A0A078JFE6	Fructose-bisphosphate aldolase	*Brassica napus*	36.8	3.4	2.0	17.5	47.5
A0A498HT56	Uncharacterized protein	*Malus domestica*	54.1	10.0	6.7	25.3	63.1
A0A2K3P1V8	S-adenosylmethionine synthase	*Trifolium pratense*	42.4	23.1	8.5	17.4	37.1
Q9LFW1	UDP-arabinopyranose mutase 2	*Arabidopsis thaliana*	10.0	2.5	1.1	3.2	16.6
P80261	NADH dehydrogenase [ubiquinone] iron–sulfur protein 3	*Solanum tuberosum*	5.9	1.8	0.7	2.0	7.5
A0A175YJ97	AAI domain-containing protein	*Daucus carota subsp. sativus*	4.3	15.5	3.6	1.5	1.9
A0A087ENY5	Phosphoglycerate kinase	*Apilactobacillus kunkeei*	50.6	57.7	16.6	22.0	25.2
A0A087EPJ5	50S ribosomal protein L4	*Apilactobacillus kunkeei*	20.7	26.0	7.3	7.4	11.6
A0A087EPM7	30S ribosomal protein S9	*Apilactobacillus kunkeei*	18.5	24.8	7.5	6.3	8.8
A0A087EQ00	Glutamine synthetase	*Apilactobacillus kunkeei*	16.2	28.4	8.0	4.6	8.7
A0A087EQA3	Threonine--tRNA ligase	*Apilactobacillus kunkeei*	14.7	20.9	5.1	4.0	9.7
A0A0M9DBL5	50S ribosomal protein L5	*Apilactobacillus kunkeei*	11.7	24.4	6.3	3.0	4.6
A0A087EPI7	DNA-directed RNA polymerase subunit beta	*Apilactobacillus kunkeei*	11.4	27.8	6.1	3.2	10.3
A0A087EQ84	Probable manganese-dependent inorganic pyrophosphatase	*Apilactobacillus kunkeei*	9.2	18.0	4.9	3.9	2.9
A0A0C3AFU8	Nitroreductase	*Apilactobacillus kunkeei*	9.0	14.7	3.8	2.6	5.7
A0A087EMN0	Glycine/betaine ABC transporter ATP-binding protein	*Apilactobacillus kunkeei*	8.6	13.6	3.2	3.1	6.0
A0A0M9D308	Catalase	*Apilactobacillus kunkeei*	6.5	18.3	4.3	2.7	3.8
A0A087EPK8	30S ribosomal protein S8	*Apilactobacillus kunkeei*	6.2	8.1	2.1	1.5	4.8
A0A0N0UVX1	Beta sliding clamp	*Apilactobacillus kunkeei*	5.5	8.3	2.1	2.0	3.0
A0A087ENA2	Aldo/keto reductase	*Apilactobacillus kunkeei*	5.4	14.6	2.7	1.1	3.8
A0A087EPK1	50S ribosomal protein L16	*Apilactobacillus kunkeei*	5.2	9.5	1.9	1.4	2.6
A0A0N0CRP1	DUF5776 domain-containing protein	*Apilactobacillus kunkeei*	4.9	14.0	6.9	9.0	0.0
A0A087EQ04	50S ribosomal protein L27	*Apilactobacillus kunkeei*	4.1	13.7	2.4	1.2	3.2
A0A0N0CQ41	6-phosphogluconate dehydrogenase, decarboxylating	*Apilactobacillus apinorum*	17.6	39.9	9.5	4.0	17.3
A0A0M9D5F1	L-lactate dehydrogenase	*Apilactobacillus apinorum*	12.5	41.2	6.3	2.1	8.0
A0A0M9D658	Glutathione reductase	*Apilactobacillus apinorum*	9.9	24.3	5.7	2.6	6.7
A0A0N0CQC3	Glutathione reductase	*Apilactobacillus apinorum*	6.8	19.4	2.5	1.2	5.3
A0A7M7RC42	Uncharacterized protein	*Apis mellifera*	43.6	15.5	11.2	48.2	10.4
A0A7M7IFB4	Glucosylceramidase	*Apis mellifera*	56.0	27.0	15.2	45.4	16.5

**Table 4:** Correlation coefficients between the number of faba bean (*Vicia faba*) pollen and the microbiota proteins number and content.

	FBP	APN	LABN	LABC
Number of faba bean pollen (FBP)	1	0.415	0.943	0.935
Aphid protein number (APN)	0.415	1	0.693	0.065
Lactic acid bacteria number (LABN)	0.943	0.693	1	0.764
Lactic acid bacteria content (LABC)	0.935	0.065	0.764	1

Note: aphid protein number (APN): *Acyrthospihon pisum*, *Aphis craccivor*, *Aphis glycines*, *Serratia symbiotica*, *Arsenophonus endosymbiont* of *aphis craccivor*; lactic acid bacteria number (LABN): *Apilactobacillus kunkeei*, *Apilactobacillus apinorum*, *Lactiplantibacillus plantarum*, *Lactiplantibacillus acidophilus*; lactic acid bacteria content (LABC): *Apilactobacillus kunkeei*, *Apilactobacillus apinorum*.

## Data Availability

The data set is available from the authors on a reasonable request.

## Supplementary Materials

The following supporting information can be downloaded at: https://www.mdpi.com/article/10.3390/life14070862/s1, Table S1: Dataset of all GO terms represented the biological process of red clover (*Trifolium pratense*) proteins annotated for different honey samples; Table S2: The number of proteins involved in biological processes of red clover proteins (*Trifolium pratense*) annotated for different honey samples; Table S3: The protein molecular functions annotation of red clover proteins (*Trifolium pratense*) of different honey samples; Table S4: Cellular components of red clover proteins (*Trifolium pratense*) identified by MS and annotated for different honey samples.

## References

[1] Hoopingarner R.H., Waller G.D., Graham J.M. (1992). Crop Pollination. The Hive and the Honeybe.

[2] Carvalho A.T., Schlindwein C. (2011). Obligate Association of an Oligolectic Bee and a Seasonal Aquatic Herb in Semi-Arid North-Eastern Brazil. Biol. J. Linn. Soc..

[3] McGregor S.E., McGregor S.E. (1976). Clover and Some Relatives. Insect Pollination of Cultivated Crop Plants.

[4] Balžekas J.A., Balžekas J. (1995). The Efficiency of the First Hybrid Generation of Different Bee Races Crossed with Caucasian Bees. Zemdirb.—Agric..

[5] Vleugels T., Amdahl H., Roldán-Ruiz I., Cnops G. (2019). Factors Underlying Seed Yield in Red Clover: Review of Current Knowledge and Perspectives. Agronomy.

[6] Free J.B. (1968). The Behaviour of Bees Visiting Runner Beans (*Phaseolus multiflorus*). J. Appl. Ecol..

[7] Vleugels T., Ceuppens B., Cnops G., Lootens P., van Parijs F.R.D., Smagghe G., Roldán-Ruiz I. (2016). Models with Only Two Predictor Variables Can Accurately Predict Seed Yield in Diploid and Tetraploid Red Clover. Euphytica.

[8] Hederström V., Rundlöf M., Birgersson G., Larsson M.C., Balkenius A., Lankinen Å. (2021). Do Plant Ploidy and Pollinator Tongue Length Interact to Cause Low Seed Yield in Red Clover?. Ecosphere.

[9] Balžekas J.A. (1995). The Most Productive Inter-Racial Bee Hybrids of the First Generation. Zemdirb.—Agric..

[10] Čeksteryte V., Navakauskiene R., Treigyte G., Jansen E., Kurtinaitiene B., Dabkevičiene G., Balžekas J. (2016). Fatty Acid Profiles of Monofloral Clover Beebread and Pollen and Proteomics of Red Clover (*Trifolium pratense*) Pollen. Biosci. Biotechnol. Biochem..

[11] Treigytė G., Zaikova I., Matuzevičius D., Čeksterytė V., Dabkevičienė G., Kurtinaitienė B., Navakauskienė R. (2014). Comparative Proteomic Analysis of Pollen of *Trifolium pratense*, *T. alexandrinum* and *T. repens*. Zemdirbyste.

[12] Smetanska I., Alharthi S.S., Selim K.A. (2021). Physicochemical, Antioxidant Capacity and Color Analysis of Six Honeys from Different Origin. J. King Saud Univ. Sci..

[13] Wang Q., Liu J., Zhu H. (2018). Genetic and Molecular Mechanisms Underlying Symbiotic Specificity in Legume-Rhizobium Interactions. Front. Plant Sci..

[14] Signorelli S., Sainz M., Tabares-da Rosa S., Monza J. (2020). The Role of Nitric Oxide in Nitrogen Fixation by Legumes. Front. Plant Sci..

[15] Brear E.M., Day D.A., Smith P.M.C. (2013). Iron: An Essential Micronutrient for the Legume-Rhizobium Symbiosis. Front. Plant Sci..

[16] Zhang D. (2013). Plant Seed-Derived Human Transferrin: Expression, Characterisation and Applications. OA Biotechnol..

[17] Ragland M., Theil E.C. (1993). Ferritin (MRNA, Protein) and Iron Concentrations during Soybean Nodule Development. Plant Mol. Biol..

[18] Strózycki P.M., Legocki A.B. (1995). Leghemoglobins from an Evolutionarily Old Legume, *Lupinus luteus*. Plant Sci..

[19] Hoppler M., Schönbächler A., Meile L., Hurrell R.F., Walczyk T. (2008). Ferritin-Iron Is Released during Boiling and In Vitro Gastric Digestion. J. Nutr..

[20] Strozycki P.M., Szczurek A., Lotocka B., Figlerowicz M., Legocki A.B. (2007). Ferritins and Nodulation in *Lupinus luteus*: Iron Management in Indeterminate Type Nodules. J. Exp. Bot..

[21] Soares S., Amaral J.S., Beatriz M., Oliveira P.P., Mafra I. (2017). A Comprehensive Review on the Main Honey Authentication Issues: Production and Origin. Compr. Rev. Food Sci. Food Saf..

[22] Siddiqui A.J., Musharraf S.G., Choudhary M.I., Rahman A.U. (2017). Application of Analytical Methods in Authentication and Adulteration of Honey. Food Chem..

[23] Čeksterytė V., Kurtinaitienė B., Jaškūnė K. (2020). The Influence of Storage Conditions on Invertase, Glucose Oxidase Activity and Free Acidity of Bee Bread and Bee-Collected Pollen Mixed with Honey and Vegetable Oils. J. Apic. Res..

[24] Louveaux J., Maurizio A., Vorwohl G. (1978). Methods of Melissopalynology. Bee World.

[25] Ruoff K., Luginbühl W., Bogdanov S., Bosset J.O., Estermann B., Ziolko T., Amadò R. (2006). Authentication of the Botanical Origin of Honey by Near-Infrared Spectroscopy. J. Agric. Food Chem..

[26] Čeksterytė V., Bliznikas S., Jaškūnė K. (2023). The Composition of Fatty Acids in Bee Pollen, Royal Jelly, Buckthorn Oil and Their Mixtures with Pollen Preserved for Storage. Foods.

[27] Barbagallo S., Cocuzza G.E.M. (2014). A Survey of the Aphid Fauna in the Italian Regions of Latium and Campania. Redia.

[28] Goławska S. (2010). Effect of Various Host-Plants on the Population Growth and Development of the Pea Aphid. J. Plant Prot. Res..

[29] Shaaban B., Seeburger V., Schroeder A., Lohaus G. (2020). Sugar, Amino Acid and Inorganic Ion Profiling of the Honeydew from Different Hemipteran Species Feeding on *Abies alba* and *Picea abies*. PLoS ONE.

[30] Recklies K., Peukert C., Kölling-Speer I., Speer K. (2021). Differentiation of Honeydew Honeys from Blossom Honeys and According to Their Botanical Origin by Electrical Conductivity and Phenolic and Sugar Spectra. J. Agric. Food Chem..

[31] Sabri A., Vandermoten S., Leroy P.D., Haubruge E., Hance T., Thonart P., De Pauw E., Francis F. (2013). Proteomic Investigation of Aphid Honeydew Reveals an Unexpected Diversity of Proteins. PLoS ONE.

[32] Čeksterytė V. (2012). Augalų Žiedadulkių, Randamų Lietuvos Meduje, Elektroninis Katalogas. [Electronic Catalog of Plant Pollen Found in Lithuanian Honey].

[33] Čeksterytė V., Kaupinis A., Jaškūnė K., Treigytė G., Navakauskienė R. (2023). Characteristics of Honey Bee Physiological Proteins Extracted from Faba Bean (*Vicia faba* L.) Honey. J. Apic. Res..

[34] Wiśniewski J., Zougman A., Nagaraj N. (2009). Universal Sample Preparation Method for Proteome Analysis. Nat. Methods.

[35] Distler U., Kuharev J., Navarro P., Levin Y., Schild H., Tenzer S. (2013). Drift Time-Specific Collision Energies Enable Deep-Coverage Data-Independent Acquisition Proteomics. Nat. Methods.

[36] Kuharev J., Navarro P., Distler U., Jahn O., Tenzer S. (2015). In-Depth Evaluation of Software Tools for Data-Independent Acquisition Based Label-Free Quantification. Proteomics.

[37] Borutinskaitė V., Treigytė G., Matuzevičius D., Čeksterytė V., Kurtinaitienė B., Serackis A., Navakauskas D., Navakauskienė R. (2019). Proteomic Studies of Honeybee- and Manually-Collected Pollen. Zemdirb.—Agric..

[38] Čeksterytė V., Borutinskaitė V., Matuzevičius D., Treigytė G., Navakauskas D., Kurtinaitienė B., Navakauskienė R. (2019). Evaluation of Proteome Profiles of *Salix* spp. Pollen and Relationship Between Glucose Oxi-Dase Activity and Pollen Content in Willow Honey. Balt For..

[39] Borutinskaitė V., Treigytė G., Matuzevičius D., Zaikova I., Čeksterytė V., Navakauskas D., Kurtinaitienė B., Navakauskienė R. (2017). Proteomic Analysis of Pollen and Blossom Honey from Rape Seed *Brassica napus* L.. J. Apic. Sci..

[40] Myhre S., Tveit H., Mollestad T., Laegreid A. (2006). Additional Gene Ontology Structure for Improved Biological Reasoning. Bioinformatics.

[41] Bargsten J.W., Severing E.I., Nap J.P., Sanchez-Perez G.F., van Dijk A.D. (2014). Biological Process Annotation of Proteins across the Plant Kingdom. Curr. Plant Biol..

[42] Chao Y., Yuan J., Li S., Jia S., Han L., Xu L. (2018). Analysis of Transcripts and Splice Isoforms in Red Clover (*Trifolium pratense* L.) by Single-Molecule Long-Read Sequencing. BMC Plant Biol..

[43] Braunschmid V., Fuerst S., Perz V., Zitzenbacher S., Hoyo J., Fernandez-Sanchez C., Tzanov T., Steinkellner G., Gruber K., Nyanhongo G.S. (2020). A Fungal Ascorbate Oxidase with Unexpected Laccase Activity. Int. J. Mol. Sci..

[44] Felton G.W., Summers C.B. (1993). Potential Role of Ascorbate Oxidase as a Plant Defense Protein against Insect Herbivory. J. Chem. Ecol..

[45] Borutinskaitė V., Treigytė G., Čeksterytė V., Kurtinaitienė B., Navakauskienė R. (2018). Proteomic Identification and Enzymatic Activity of Buckwheat (*Fagopyrum esculentum*) Honey Based on Different Assays. J. Food Nutr. Res..

[46] Kretavičius J., Kurtinaitienė B., Račys J., Čeksterytė V. (2010). Inactivation of Glucose Oxidase during Heat-Treatment de-Crystallization of Honey. Zemdirb.—Agric..

[47] Mockaitis K., Estelle M. (2004). Integrating Transcriptional Controls for Plant Cell Expansion. Genome Biol..

[48] Luschnig C. (2001). Auxin Transport: Why Plants like to Think BIG. Curr. Biol..

[49] Mohanta T.K., Khan A., Hashem A., Abd_Allah E.F., Al-Harrasi A. (2019). The Molecular Mass and Isoelectric Point of Plant Proteomes. BMC Genom..

[50] Grillo M.A., Colombatto S. (2008). S-Adenosylmethionine and Its Products. Amino Acids.

[51] Markham G.D., Pajares M.A. (2009). Structure-Function Relationships in Methionine Adenosyltransferases. Cell. Mol. Life Sci..

[52] Bakshi A., Shemansky J.M., Chang C., Binder B.M. (2015). History of Research on the Plant Hormone Ethylene. J. Plant Growth Regul..

[53] Zeng Z., Zeng X., Guo Y., Wu Z., Cai Z., Pan D. (2022). Determining the Role of UTP-Glucose-1-Phosphate Uridylyltransferase (GalU) in Improving the Resistance of *Lactobacillus acidophilus* NCFM to Freeze-Drying. Foods.

[54] Coleman H.D., Ellis D.D., Gilbert M., Mansfield S.D. (2006). Up-Regulation of Sucrose Synthase and UDP-Glucose Pyrophosphorylase Impacts Plant Growth and Metabolism. Plant Biotechnol. J..

[55] Kleczkowski L.A., Geisler M., Ciereszko I., Johansson H. (2004). UDP-Glucose Pyrophosphorylase. An Old Protein with New Tricks. Plant Physiol..

[56] Thoden J.B., Holden H.M. (2007). The Molecular Architecture of Glucose-1-Phosphate Uridylyltransferase. Protein Sci..

[57] Chua T.K., Bujnicki J.M., Tan T.-C., Huynh F., Patel B.K., Sivaraman J. (2008). The Structure of Sucrose Phosphate Synthase from *Halothermothrix orenii* Reveals Its Mechanism of Action and Binding Mode. Plant Cell.

[58] Hasunuma T., Harada K., Miyazawa S.-I., Kondo A., Fukusaki E., Miyake C. (2010). Metabolic Turnover Analysis by a Combination of in Vivo 13C-Labelling from 13CO2 and Metabolic Profiling with CE-MS/MS Reveals Rate-Limiting Steps of the C3 Photosynthetic Pathway in *Nicotiana tabacum* Leaves. J. Exp. Bot..

[59] Decker D., Kleczkowski L.A. (2019). UDP-Sugar Producing Pyrophosphorylases: Distinct and Essential Enzymes with Overlapping Substrate Specificities, Providing de Novo Precursors for Glycosylation Reactions. Front. Plant Sci..

[60] Bar-Peled M., O’Neill M.A. (2011). Plant Nucleotide Sugar Formation, Interconversion, and Salvage by Sugar Recycling. Annu. Rev. Plant Biol..

[61] Orellana A., Moraga C., Araya M., Moreno A. (2016). Overview of Nucleotide Sugar Transporter Gene Family Functions Across Multiple Species. J. Mol. Biol..

[62] Liu D., Wang L., Liu C., Song X., He S., Zhai H., Liu Q. (2014). An Ipomoea Batatas Iron-Sulfur Cluster Scaffold Protein Gene, IbNFU1, Is Involved in Salt Tolerance. PLoS ONE.

[63] Olson J.W., Agar J.N., Johnson M.K., Maier R.J. (2000). Characterization of the NifU and NifS Fe−S Cluster Formation Proteins Essential for Viability in *Helicobacter pylori*. Biochemistry.

[64] Yuvaniyama P., Agar J.N., Cash V.L., Johnson M.K., Dean D.R. (2000). NifS-Directed Assembly of a Transient [2Fe-2S] Cluster within the NifU Protein. Proc. Natl. Acad. Sci. USA.

[65] Hwang D.M., Dempsey A., Tan K.-T., Liew C.-C. (1996). A Modular Domain of NifU, a Nitrogen Fixation Cluster Protein, Is Highly Conserved in Evolution. J. Mol. Evol..

[66] Lee E.H., Lee K., Hwang K.Y. (2014). Structural Characterization and Comparison of the Large Subunits of IPM Isomerase and Homoaconitase from *Methanococcus jannaschii*. Acta Crystallogr. D Biol. Crystallogr..

[67] Dunn M.F. (1998). Tricarboxylic Acid Cycle and Anaplerotic Enzymes in Rhizobia. FEMS Microbiol. Rev..

[68] Resendis-Antonio O., Hernández M., Salazar E., Contreras S., Batallar G.M., Mora Y., Encarnación S. (2011). Systems Biology of Bacterial Nitrogen Fixation: High-Throughput Technology and Its Integrative Description with Constraint-Based Modeling. BMC Syst. Biol..

[69] Shaaban B., Seeburger V., Schroeder A., Lohaus G. (2021). Suitability of Sugar, Amino Acid, and Inorganic Ion Compositions to Distinguish Fir and Spruce Honey. Eur. Food Res. Technol..

[70] Capinera J.L. (2001). Green Peach Aphid, Myzus Persicae (Sulzer) (Insecta: Hemiptera: Aphididae).

[71] Sandhi R.K., Reddy G.V.P. (2020). Biology, Ecology, and Management Strategies for Pea Aphid (Hemiptera: Aphididae) in Pulse Crops. J. Integr. Pest Manag..

[72] Ashby J.W., Fletcher J.D., Farrell J.A.K., Stufkens M.R. (1982). Observations on Host Preferences and Epidemiology of Aphid Species Associated with Legume Crops. N. Z. J. Agric. Res..

[73] Paudel S., Bechinski E.J., Stokes B.S., Pappu H.R., Eigenbrode D.S. (2018). Deriving Economic Models for Pea Aphid (Hemiptera: Aphididae) as a Direct-Pest and a Virus-Vector on Commercial Lentils. J. Econ. Entomol..

[74] Blackman R.L., Eastop V.F. (2000). Aphids on the World’s Crops. An Identification and Information Guide.

[75] Osiadacz B., Hałaj R., Chmura D. (2017). Biodiversity—Economy or Ecology? Long-Term Study of Changes in the Biodiversity of Aphids Living in Steppe-like Grasslands in Central Europe. Eur. J. Entomol..

[76] Shigenobu S., Hattori H.W.M., Sakaki Y., Ishikawa H. (2000). Genome Sequence of the Endocellular Bacterial Symbiont of Aphids *Buchnera* sp. APS. Nature.

[77] Hoy M.A. (2013). Genetic Systems, Genome Evolution, and Genetic Control of Embryonic Development in Insects. Insect Molecular Genetics: An Introduction to Principles and Applications.

[78] Olofsson T.C., Butler È., Markowicz P., Lindholm C., Larsson L., Vásquez A. (2016). Lactic Acid Bacterial Symbionts in Honeybees—An Unknown Key to Honey’s Antimicrobial and Therapeutic Activities. Int. Wound J..

[79] Olofsson T.C., Vásquez A. (2008). Detection and Identification of a Novel Lactic Acid Bacterial Flora within the Honey Stomach of the Honeybee *Apis mellifera*. Curr. Microbiol..

[80] De Jesus L., Aburjaile F., Sousa T., Felice A., Soares S., Alcantara L., Azevedo V. (2022). Genomic Characterization of *Lactobacillus delbrueckii* Strains with Probiotics Properties. Front. Bioinform..

[81] Gustaw K., Michalak M., Polak-Berecka M., Waśko A. (2018). Isolation and Characterization of a New Fructophilic *Lactobacillus plantarum* FPL Strain from Honeydew. Ann. Microbiol..

[82] Iorizzo M., Pannella G., Lombardi S., Ganassi S., Testa B., Succi M., Sorrentino E., Petrarca S., De Cristofaro A., Coppola R. (2020). Inter- and Intra-Species Diversity of Lactic Acid Bacteria in *Apis mellifera* Ligustica Colonies. Microorganisms.

[83] Endo A., Futagawa-Endo Y., Dicks L. (2009). Isolation and Characterization of Fructophilic Lactic Acid Bacteria from Fructose-Rich Niches. Syst. Appl. Microbiol..

[84] Endo A., Salminen S. (2013). Honeybees and Beehives Are Rich Sources for Fructophilic Lactic Acid Bacteria. Syst. Appl. Microbiol..

[85] Filannino P., Di Cagno R., Tlais A.Z.A., Cantatore V., Gobbetti M. (2019). Fructose-Rich Niches Traced the Evolution of Lactic Acid Bacteria toward Fructophilic Species. Crit. Rev. Microbiol..

