# Supplementary material for: Composition of Proteins Associated with Red Clover (Trifolium pratense) and the Microbiota Identified in Honey
Source: Life (Basel). 2024 Jul 10;14(7):862. doi: 10.3390/life14070862 (PMC11278118; doi:10.3390/life14070862)
Supplement: Supplementary file 1 [file life-14-00862-s001.zip › Table S1.pdf]

Supplementary Table S1. Dataset of all GO terms represented the biological process of red clover (*Trifolium pratense*) proteins annotated for different honey samples.

GO Category	GO Slim Identifier	GO Slim Term Name	Uniprot accession number	GO Identifier	GO Term Name
C	GO:0005575	cellular_component	A0A2K3NMQ3	GO:0030659	cytoplasmic vesicle membrane
C	GO:0005575	cellular_component	A0A2K3JM62	GO:0015935	small ribosomal subunit
C	GO:0005575	cellular_component	A0A2K3M1W9	GO:0015935	small ribosomal subunit
C	GO:0005575	cellular_component	A0A2K3NMQ3	GO:0016020	membrane
C	GO:0005575	cellular_component	A0A2K3NV46	GO:0016020	membrane
C	GO:0005575	cellular_component	A0A2K3P8U8	GO:0016020	membrane
C	GO:0005575	cellular_component	A0A2K3NMQ3	GO:0005905	clathrin-coated pit
C	GO:0005575	cellular_component	A0A2K3NKS8	GO:0000123	histone acetyltransferase complex
C	GO:0005575	cellular_component	A0A2K3NV46	GO:0016021	integral component of membrane
C	GO:0005575	cellular_component	A0A2K3P8U8	GO:0016021	integral component of membrane
C	GO:0005575	cellular_component	A0A2K3NQ11	GO:0005759	mitochondrial matrix
C	GO:0005575	cellular_component	A0A2K3NMQ3	GO:0071439	clathrin complex
C	GO:0005575	cellular_component	A0A2K3P6Y1	GO:0071439	clathrin complex
C	GO:0005575	cellular_component	A0A2K3NLX6	GO:0033180	proton-transporting V-type ATPase, V1 domain
C	GO:0005575	cellular_component	A0A2K3NMQ3	GO:0030130	clathrin coat of trans-Golgi network vesicle
C	GO:0005575	cellular_component	A0A2K3NMQ3	GO:0030132	clathrin coat of coated pit
C	GO:0005622	intracellular	A0A2K3NMQ3	GO:0030659	cytoplasmic vesicle membrane
C	GO:0005622	intracellular	A0A2K3JM62	GO:0015935	small ribosomal subunit
C	GO:0005622	intracellular	A0A2K3M1W9	GO:0015935	small ribosomal subunit
C	GO:0005622	intracellular	A0A2K3NKS8	GO:0000123	histone acetyltransferase complex
C	GO:0005622	intracellular	A0A2K3NQ11	GO:0005759	mitochondrial matrix
C	GO:0005622	intracellular	A0A2K3NMQ3	GO:0071439	clathrin complex
C	GO:0005622	intracellular	A0A2K3P6Y1	GO:0071439	clathrin complex
C	GO:0005622	intracellular	A0A2K3NMQ3	GO:0030130	clathrin coat of trans-Golgi network vesicle
C	GO:0005622	intracellular	A0A2K3NMQ3	GO:0030132	clathrin coat of coated pit
C	GO:0005623	cell	A0A2K3NMQ3	GO:0030659	cytoplasmic vesicle membrane
C	GO:0005623	cell	A0A2K3JM62	GO:0015935	small ribosomal subunit
C	GO:0005623	cell	A0A2K3M1W9	GO:0015935	small ribosomal subunit
C	GO:0005623	cell	A0A2K3NMQ3	GO:0005905	clathrin-coated pit
C	GO:0005623	cell	A0A2K3NKS8	GO:0000123	histone acetyltransferase complex
C	GO:0005623	cell	A0A2K3NQ11	GO:0005759	mitochondrial matrix
C	GO:0005623	cell	A0A2K3NMQ3	GO:0071439	clathrin complex
C	GO:0005623	cell	A0A2K3P6Y1	GO:0071439	clathrin complex
C	GO:0005623	cell	A0A2K3NMQ3	GO:0030130	clathrin coat of trans-Golgi network vesicle
C	GO:0005623	cell	A0A2K3NMQ3	GO:0030132	clathrin coat of coated pit

C	GO:0005634	nucleus	A0A2K3NKS8	GO:0005634	nucleus
C	GO:0005634	nucleus	A0A2K3NKS8	GO:0000123	histone acetyltransferase complex
C	GO:0005654	nucleoplasm	A0A2K3NKS8	GO:0000123	histone acetyltransferase complex
C	GO:0005737	cytoplasm	A0A2K3MW20	GO:0005737	cytoplasm
C	GO:0005737	cytoplasm	A0A2K3NMQ3	GO:0030659	cytoplasmic vesicle membrane
C	GO:0005737	cytoplasm	A0A2K3JM62	GO:0015935	small ribosomal subunit
C	GO:0005737	cytoplasm	A0A2K3M1W9	GO:0015935	small ribosomal subunit
C	GO:0005737	cytoplasm	A0A2K3NQ11	GO:0005759	mitochondrial matrix
C	GO:0005737	cytoplasm	A0A2K3NMQ3	GO:0071439	clathrin complex
C	GO:0005737	cytoplasm	A0A2K3P6Y1	GO:0071439	clathrin complex
C	GO:0005737	cytoplasm	A0A2K3NMQ3	GO:0030130	clathrin coat of trans-Golgi network vesicle
C	GO:0005737	cytoplasm	A0A2K3NMQ3	GO:0030132	clathrin coat of coated pit
C	GO:0005739	mitochondrion	A0A2K3NQ11	GO:0005739	mitochondrion
C	GO:0005739	mitochondrion	A0A2K3NQ11	GO:0005759	mitochondrial matrix
C	GO:0005794	Golgi apparatus	A0A2K3NMQ3	GO:0030130	clathrin coat of trans-Golgi network vesicle
C	GO:0005840	ribosome	A0A2K3JM62	GO:0015935	small ribosomal subunit
C	GO:0005840	ribosome	A0A2K3M1W9	GO:0015935	small ribosomal subunit
C	GO:0005840	ribosome	A0A2K3JM62	GO:0005840	ribosome
C	GO:0005840	ribosome	A0A2K3LKQ2	GO:0005840	ribosome
C	GO:0005840	ribosome	A0A2K3LNN3	GO:0005840	ribosome
C	GO:0005840	ribosome	A0A2K3LUX0	GO:0005840	ribosome
C	GO:0005840	ribosome	A0A2K3M1W9	GO:0005840	ribosome
C	GO:0005840	ribosome	A0A2K3MF29	GO:0005840	ribosome
C	GO:0005840	ribosome	A0A2K3PKP0	GO:0005840	ribosome
C	GO:0005886	plasma membrane	A0A2K3NMQ3	GO:0030132	clathrin coat of coated pit
C	GO:0005886	plasma membrane	A0A2K3P8U8	GO:0005886	plasma membrane
C	GO:0031410	cytoplasmic vesicle	A0A2K3NMQ3	GO:0030659	cytoplasmic vesicle membrane
C	GO:0031410	cytoplasmic vesicle	A0A2K3NMQ3	GO:0031410	cytoplasmic vesicle
C	GO:0031410	cytoplasmic vesicle	A0A2K3NMQ3	GO:0030130	clathrin coat of trans-Golgi network vesicle
C	GO:0032991	protein-containing complex	A0A2K3JM62	GO:0015935	small ribosomal subunit
C	GO:0032991	protein-containing complex	A0A2K3M1W9	GO:0015935	small ribosomal subunit
C	GO:0032991	protein-containing complex	A0A2K3NKS8	GO:0000123	histone acetyltransferase complex
C	GO:0032991	protein-containing complex	A0A2K3NMQ3	GO:0071439	clathrin complex
C	GO:0032991	protein-containing complex	A0A2K3P6Y1	GO:0071439	clathrin complex
C	GO:0032991	protein-containing complex	A0A2K3NLX6	GO:0033180	proton-transporting V-type ATPase, V1 domain
C	GO:0032991	protein-containing complex	A0A2K3NMQ3	GO:0030130	clathrin coat of trans-Golgi network vesicle
C	GO:0032991	protein-containing complex	A0A2K3NMQ3	GO:0030132	clathrin coat of coated pit
C	GO:0043226	organelle	A0A2K3NMQ3	GO:0030659	cytoplasmic vesicle membrane
C	GO:0043226	organelle	A0A2K3JM62	GO:0015935	small ribosomal subunit
C	GO:0043226	organelle	A0A2K3M1W9	GO:0015935	small ribosomal subunit

C	GO:0043226	organelle	A0A2K3NKS8	GO:0000123	histone acetyltransferase complex
C	GO:0043226	organelle	A0A2K3NQ11	GO:0005759	mitochondrial matrix
C	GO:0043226	organelle	A0A2K3NMQ3	GO:0030130	clathrin coat of trans-Golgi network vesicle
F	GO:0003674	molecular_function	A0A2K3NRS2	GO:0016773	phosphotransferase activity, alcohol group as acceptor
F	GO:0003674	molecular_function	A0A2K3PJL4	GO:0016773	phosphotransferase activity, alcohol group as acceptor
F	GO:0003674	molecular_function	A0A2K3MUC7	GO:0051287	NAD binding
F	GO:0003674	molecular_function	A0A2K3PPZ5	GO:0051287	NAD binding
F	GO:0003674	molecular_function	A0A2K3P3K7	GO:0003871	5-methyltetrahydropteroyltriglutamate-homocysteine S-methyltransferase activity
F	GO:0003674	molecular_function	A0A2K3NMF1	GO:0004372	glycine hydroxymethyltransferase activity
F	GO:0003674	molecular_function	A0A2K3NU12	GO:0051536	iron-sulfur cluster binding
F	GO:0003674	molecular_function	A0A2K3NKS8	GO:0004674	protein serine/threonine kinase activity
F	GO:0003674	molecular_function	A0A2K3NMF1	GO:0030170	pyridoxal phosphate binding
F	GO:0003674	molecular_function	A0A2K3NLX6	GO:0046961	proton-transporting ATPase activity, rotational mechanism
F	GO:0003674	molecular_function	A0A2K3LNL7	GO:0000166	nucleotide binding
F	GO:0003674	molecular_function	A0A2K3MW20	GO:0000166	nucleotide binding
F	GO:0003674	molecular_function	A0A2K3N3T6	GO:0000166	nucleotide binding
F	GO:0003674	molecular_function	A0A2K3NCQ3	GO:0000166	nucleotide binding
F	GO:0003674	molecular_function	A0A2K3NKS8	GO:0000166	nucleotide binding
F	GO:0003674	molecular_function	A0A2K3P1V8	GO:0000166	nucleotide binding
F	GO:0003674	molecular_function	A0A2K3P8U8	GO:0000166	nucleotide binding
F	GO:0003674	molecular_function	A0A2K3NLG1	GO:0004842	ubiquitin-protein transferase activity
F	GO:0003674	molecular_function	A0A2K3PPN3	GO:0050662	coenzyme binding
F	GO:0003674	molecular_function	A0A2K3NU12	GO:0051539	4 iron, 4 sulfur cluster binding
F	GO:0003674	molecular_function	A0A2K3PGB1	GO:0030246	carbohydrate binding
F	GO:0003674	molecular_function	A0A2K3MUC7	GO:0016787	hydrolase activity
F	GO:0003674	molecular_function	A0A2K3MW20	GO:0016787	hydrolase activity
F	GO:0003674	molecular_function	A0A2K3N3T6	GO:0016787	hydrolase activity
F	GO:0003674	molecular_function	A0A2K3PGB1	GO:0016787	hydrolase activity
F	GO:0003674	molecular_function	A0A2K3P1V8	GO:0004478	methionine adenosyltransferase activity
F	GO:0003674	molecular_function	A0A2K3MUC7	GO:0004013	adenosylhomocysteinase activity
F	GO:0003674	molecular_function	A0A2K3P5Y0	GO:0030976	thiamine pyrophosphate binding
F	GO:0003674	molecular_function	A0A2K3NCQ3	GO:0043531	ADP binding
F	GO:0003674	molecular_function	A0A2K3PQG8	GO:0019904	protein domain specific binding
F	GO:0003674	molecular_function	A0A2K3P8U8	GO:0008553	proton-exporting ATPase activity, phosphorylative mechanism
F	GO:0003674	molecular_function	A0A2K3MUW7	GO:0016776	phosphotransferase activity, phosphate group as acceptor
F	GO:0003674	molecular_function	A0A2K3MUW7	GO:0004017	adenylate kinase activity
F	GO:0003674	molecular_function	A0A2K3NMQ3	GO:0032051	clathrin light chain binding
F	GO:0003674	molecular_function	A0A2K3P6Y1	GO:0032051	clathrin light chain binding
F	GO:0003674	molecular_function	A0A2K3LNL7	GO:0005524	ATP binding

F	GO:0003674	molecular_function	A0A2K3MA51	GO:0005524	ATP binding
F	GO:0003674	molecular_function	A0A2K3MUW7	GO:0005524	ATP binding
F	GO:0003674	molecular_function	A0A2K3MW20	GO:0005524	ATP binding
F	GO:0003674	molecular_function	A0A2K3N3T6	GO:0005524	ATP binding
F	GO:0003674	molecular_function	A0A2K3NCQ3	GO:0005524	ATP binding
F	GO:0003674	molecular_function	A0A2K3NKS8	GO:0005524	ATP binding
F	GO:0003674	molecular_function	A0A2K3NLX6	GO:0005524	ATP binding
F	GO:0003674	molecular_function	A0A2K3P1V8	GO:0005524	ATP binding
F	GO:0003674	molecular_function	A0A2K3P8U8	GO:0005524	ATP binding
F	GO:0003674	molecular_function	A0A2K3PN04	GO:0005524	ATP binding
F	GO:0003674	molecular_function	A0A2K3MUW7	GO:0016740	transferase activity
F	GO:0003674	molecular_function	A0A2K3NKS8	GO:0016740	transferase activity
F	GO:0003674	molecular_function	A0A2K3NLG1	GO:0016740	transferase activity
F	GO:0003674	molecular_function	A0A2K3NMF1	GO:0016740	transferase activity
F	GO:0003674	molecular_function	A0A2K3NWT6	GO:0016740	transferase activity
F	GO:0003674	molecular_function	A0A2K3P1V8	GO:0016740	transferase activity
F	GO:0003674	molecular_function	A0A2K3P3K7	GO:0016740	transferase activity
F	GO:0003674	molecular_function	A0A2K3NK64	GO:0003824	catalytic activity
F	GO:0003674	molecular_function	A0A2K3NMF1	GO:0003824	catalytic activity
F	GO:0003674	molecular_function	A0A2K3NQ11	GO:0003824	catalytic activity
F	GO:0003674	molecular_function	A0A2K3PCD9	GO:0003824	catalytic activity
F	GO:0003674	molecular_function	A0A2K3PPN3	GO:0003824	catalytic activity
F	GO:0003674	molecular_function	A0A2K3MW20	GO:0036402	proteasome-activating ATPase activity
F	GO:0003723	RNA binding	A0A2K3M1W9	GO:0003723	RNA binding
F	GO:0003735	structural constituent of ribosome	A0A2K3JM62	GO:0003735	structural constituent of ribosome
F	GO:0003735	structural constituent of ribosome	A0A2K3LKQ2	GO:0003735	structural constituent of ribosome
F	GO:0003735	structural constituent of ribosome	A0A2K3LNN3	GO:0003735	structural constituent of ribosome
F	GO:0003735	structural constituent of ribosome	A0A2K3LUX0	GO:0003735	structural constituent of ribosome
F	GO:0003735	structural constituent of ribosome	A0A2K3M1W9	GO:0003735	structural constituent of ribosome
F	GO:0003735	structural constituent of ribosome	A0A2K3MF29	GO:0003735	structural constituent of ribosome
F	GO:0005198	structural molecule activity	A0A2K3NMQ3	GO:0005198	structural molecule activity
F	GO:0008168	methyltransferase activity	A0A2K3P3K7	GO:0003871	5-methyltetrahydropteroyltriglutamate-homocysteine S-methyltransferase activity
F	GO:0008168	methyltransferase activity	A0A2K3NMF1	GO:0008168	methyltransferase activity
F	GO:0008168	methyltransferase activity	A0A2K3P3K7	GO:0008168	methyltransferase activity
F	GO:0008233	peptidase activity	A0A2K3MW20	GO:0008233	peptidase activity
F	GO:0016301	kinase activity	A0A2K3NKS8	GO:0004674	protein serine/threonine kinase activity
F	GO:0016301	kinase activity	A0A2K3MUW7	GO:0016301	kinase activity
F	GO:0016301	kinase activity	A0A2K3NKS8	GO:0016301	kinase activity

F	GO:0016301	kinase activity	A0A2K3NRS2	GO:0016301	kinase activity
F	GO:0016301	kinase activity	A0A2K3PJL4	GO:0016301	kinase activity
F	GO:0016301	kinase activity	A0A2K3MUW7	GO:0004017	adenylate kinase activity
F	GO:0016301	kinase activity	A0A2K3MUW7	GO:0019205	nucleobase-containing compound kinase activity
F	GO:0016491	oxidoreductase activity	A0A2K3PPZ5	GO:0008863	formate dehydrogenase (NAD+) activity
F	GO:0016491	oxidoreductase activity	A0A2K3PCD9	GO:0015930	glutamate synthase activity
F	GO:0016491	oxidoreductase activity	A0A2K3PPZ5	GO:0016616	oxidoreductase activity, acting on the CH-OH group of donors, NAD or NADP as acceptor
F	GO:0016491	oxidoreductase activity	A0A2K3PCD9	GO:0016638	oxidoreductase activity, acting on the CH-NH2 group of donors
F	GO:0016491	oxidoreductase activity	A0A2K3NQ11	GO:0004739	pyruvate dehydrogenase (acetyl-transferring) activity
F	GO:0016491	oxidoreductase activity	A0A2K3P5Y0	GO:0016624	oxidoreductase activity, acting on the aldehyde or oxo group of donors, disulfide as acceptor
F	GO:0016491	oxidoreductase activity	A0A2K3NL25	GO:0016491	oxidoreductase activity
F	GO:0016491	oxidoreductase activity	A0A2K3NQ11	GO:0016491	oxidoreductase activity
F	GO:0016491	oxidoreductase activity	A0A2K3PPZ5	GO:0016491	oxidoreductase activity
F	GO:0016491	oxidoreductase activity	A0A2K3P5Y0	GO:0004591	oxoglutarate dehydrogenase (succinyl-transferring) activity
F	GO:0016765	transferase activity, transferring alkyl or aryl (other than methyl) groups	A0A2K3P1V8	GO:0004478	methionine adenosyltransferase activity
F	GO:0016779	nucleotidyltransferase activity	A0A2K3NWT6	GO:0016779	nucleotidyltransferase activity
F	GO:0016779	nucleotidyltransferase activity	A0A2K3NWT6	GO:0070569	uridylyltransferase activity
F	GO:0016779	nucleotidyltransferase activity	A0A2K3NWT6	GO:0003983	UTP:glucose-1-phosphate uridylyltransferase activity
F	GO:0016798	hydrolase activity, acting on glycosyl bonds	A0A2K3PGB1	GO:0004553	hydrolase activity, hydrolyzing O-glycosyl compounds
F	GO:0016798	hydrolase activity, acting on glycosyl bonds	A0A2K3PGB1	GO:0016798	hydrolase activity, acting on glycosyl bonds
F	GO:0016798	hydrolase activity, acting on glycosyl bonds	A0A2K3PGB1	GO:0004565	beta-galactosidase activity
F	GO:0016829	lyase activity	A0A2K3NU12	GO:0003994	aconitate hydratase activity
F	GO:0016829	lyase activity	A0A2K3NU12	GO:0016829	lyase activity
F	GO:0016853	isomerase activity	A0A2K3PGD6	GO:0016866	intramolecular transferase activity
F	GO:0016853	isomerase activity	A0A2K3PKL4	GO:0016866	intramolecular transferase activity
F	GO:0016874	ligase activity	A0A2K3NLG1	GO:0016874	ligase activity
F	GO:0016887	ATPase activity	A0A2K3NLX6	GO:0046961	proton-transporting ATPase activity, rotational mechanism
F	GO:0016887	ATPase activity	A0A2K3P8U8	GO:0008553	proton-exporting ATPase activity, phosphorylative mechanism
F	GO:0016887	ATPase activity	A0A2K3MW20	GO:0036402	proteasome-activating ATPase activity
F	GO:0019843	rRNA binding	A0A2K3M1W9	GO:0019843	rRNA binding

F	GO:0022857	transmembrane transporter activity	A0A2K3NLX6	GO:0046961	proton-transporting ATPase activity, rotational mechanism
F	GO:0022857	transmembrane transporter activity	A0A2K3NV46	GO:0022857	transmembrane transporter activity
F	GO:0022857	transmembrane transporter activity	A0A2K3P8U8	GO:0008553	proton-exporting ATPase activity, phosphorylative mechanism
F	GO:0043167	ion binding	A0A2K3NL22	GO:0008270	zinc ion binding
F	GO:0043167	ion binding	A0A2K3P3K7	GO:0008270	zinc ion binding
F	GO:0043167	ion binding	A0A2K3NMF1	GO:0030170	pyridoxal phosphate binding
F	GO:0043167	ion binding	A0A2K3NL25	GO:0005507	copper ion binding
F	GO:0043167	ion binding	A0A2K3P5Y0	GO:0030976	thiamine pyrophosphate binding
F	GO:0043167	ion binding	A0A2K3NCQ3	GO:0043531	ADP binding
F	GO:0043167	ion binding	A0A2K3LNL7	GO:0005524	ATP binding
F	GO:0043167	ion binding	A0A2K3MA51	GO:0005524	ATP binding
F	GO:0043167	ion binding	A0A2K3MUW7	GO:0005524	ATP binding
F	GO:0043167	ion binding	A0A2K3MW20	GO:0005524	ATP binding
F	GO:0043167	ion binding	A0A2K3N3T6	GO:0005524	ATP binding
F	GO:0043167	ion binding	A0A2K3NCQ3	GO:0005524	ATP binding
F	GO:0043167	ion binding	A0A2K3NKS8	GO:0005524	ATP binding
F	GO:0043167	ion binding	A0A2K3NLX6	GO:0005524	ATP binding
F	GO:0043167	ion binding	A0A2K3P1V8	GO:0005524	ATP binding
F	GO:0043167	ion binding	A0A2K3P8U8	GO:0005524	ATP binding
F	GO:0043167	ion binding	A0A2K3PN04	GO:0005524	ATP binding
F	GO:0043167	ion binding	A0A2K3NK64	GO:0046872	metal ion binding
F	GO:0043167	ion binding	A0A2K3NL22	GO:0046872	metal ion binding
F	GO:0043167	ion binding	A0A2K3P1V8	GO:0046872	metal ion binding
F	GO:0051082	unfolded protein binding	A0A2K3PN04	GO:0051082	unfolded protein binding
P	GO:0005975	carbohydrate metabolic process	A0A2K3NQ11	GO:0006096	glycolytic process
P	GO:0005975	carbohydrate metabolic process	A0A2K3PGB1	GO:0005975	carbohydrate metabolic process
P	GO:0006091	generation of precursor metabolites and energy	A0A2K3NQ11	GO:0006096	glycolytic process
P	GO:0006091	generation of precursor metabolites and energy	A0A2K3P5Y0	GO:0006099	tricarboxylic acid cycle
P	GO:0006412	translation	A0A2K3JM62	GO:0006412	translation
P	GO:0006412	translation	A0A2K3LKQ2	GO:0006412	translation
P	GO:0006412	translation	A0A2K3LNN3	GO:0006412	translation
P	GO:0006412	translation	A0A2K3LUX0	GO:0006412	translation
P	GO:0006412	translation	A0A2K3M1W9	GO:0006412	translation
P	GO:0006412	translation	A0A2K3MF29	GO:0006412	translation
P	GO:0006457	protein folding	A0A2K3PN04	GO:0006457	protein folding
P	GO:0006464	cellular protein modification process	A0A2K3NKS8	GO:0016573	histone acetylation
P	GO:0006464	cellular protein modification process	A0A2K3NKS8	GO:0006468	protein phosphorylation

P	GO:0006464	cellular protein modification process	A0A2K3NLG1	GO:0016567	protein ubiquitination
P	GO:0006520	cellular amino acid metabolic process	A0A2K3NMF1	GO:0019264	glycine biosynthetic process from serine
P	GO:0006520	cellular amino acid metabolic process	A0A2K3P3K7	GO:0008652	cellular amino acid biosynthetic process
P	GO:0006520	cellular amino acid metabolic process	A0A2K3MUC7	GO:0019510	S-adenosylhomocysteine catabolic process
P	GO:0006520	cellular amino acid metabolic process	A0A2K3P3K7	GO:0009086	methionine biosynthetic process
P	GO:0006520	cellular amino acid metabolic process	A0A2K3PCD9	GO:0006537	glutamate biosynthetic process
P	GO:0006790	sulfur compound metabolic process	A0A2K3P1V8	GO:0006556	S-adenosylmethionine biosynthetic process
P	GO:0006790	sulfur compound metabolic process	A0A2K3MUC7	GO:0019510	S-adenosylhomocysteine catabolic process
P	GO:0006790	sulfur compound metabolic process	A0A2K3P3K7	GO:0009086	methionine biosynthetic process
P	GO:0006790	sulfur compound metabolic process	A0A2K3NQ11	GO:0006086	acetyl-CoA biosynthetic process from pyruvate
P	GO:0006810	transport	A0A2K3NLX6	GO:1902600	proton transmembrane transport
P	GO:0006810	transport	A0A2K3P8U8	GO:0120029	proton export across plasma membrane
P	GO:0006810	transport	A0A2K3NMQ3	GO:0006886	intracellular protein transport
P	GO:0006810	transport	A0A2K3P6Y1	GO:0006886	intracellular protein transport
P	GO:0006810	transport	A0A2K3P8U8	GO:0006811	ion transport
P	GO:0006810	transport	A0A2K3NLX6	GO:0015991	ATP hydrolysis coupled proton transport
P	GO:0007165	signal transduction	A0A2K3NCQ3	GO:0007165	signal transduction
P	GO:0008150	biological_process	A0A2K3PGD6	GO:0071669	plant-type cell wall organization or biogenesis
P	GO:0008150	biological_process	A0A2K3PKL4	GO:0071669	plant-type cell wall organization or biogenesis
P	GO:0008150	biological_process	A0A2K3NL25	GO:0055114	oxidation-reduction process
P	GO:0008150	biological_process	A0A2K3NQ11	GO:0055114	oxidation-reduction process
P	GO:0008150	biological_process	A0A2K3P5Y0	GO:0055114	oxidation-reduction process
P	GO:0008150	biological_process	A0A2K3PCD9	GO:0055114	oxidation-reduction process
P	GO:0008150	biological_process	A0A2K3PPZ5	GO:0055114	oxidation-reduction process
P	GO:0008150	biological_process	A0A2K3NLX6	GO:1902600	proton transmembrane transport
P	GO:0008150	biological_process	A0A2K3NQ11	GO:0006096	glycolytic process
P	GO:0008150	biological_process	A0A2K3P8U8	GO:0120029	proton export across plasma membrane
P	GO:0008150	biological_process	A0A2K3NMF1	GO:0019264	glycine biosynthetic process from serine
P	GO:0008150	biological_process	A0A2K3NMF1	GO:0032259	methylation
P	GO:0008150	biological_process	A0A2K3P3K7	GO:0032259	methylation
P	GO:0008150	biological_process	A0A2K3MUW7	GO:0006139	nucleobase-containing compound metabolic process
P	GO:0008150	biological_process	A0A2K3NKS8	GO:0016573	histone acetylation
P	GO:0008150	biological_process	A0A2K3P3K7	GO:0008652	cellular amino acid biosynthetic process
P	GO:0008150	biological_process	A0A2K3MW20	GO:0006508	proteolysis
P	GO:0008150	biological_process	A0A2K3NMF1	GO:0035999	tetrahydrofolate interconversion
P	GO:0008150	biological_process	A0A2K3MUW7	GO:0046940	nucleoside monophosphate phosphorylation

P	GO:0008150	biological_process	A0A2K3NLX6	GO:0046034	ATP metabolic process
P	GO:0008150	biological_process	A0A2K3P5Y0	GO:0006099	tricarboxylic acid cycle
P	GO:0008150	biological_process	A0A2K3PGB1	GO:0008152	metabolic process
P	GO:0008150	biological_process	A0A2K3P1V8	GO:0006556	S-adenosylmethionine biosynthetic process
P	GO:0008150	biological_process	A0A2K3MUC7	GO:0006730	one-carbon metabolic process
P	GO:0008150	biological_process	A0A2K3NMF1	GO:0006730	one-carbon metabolic process
P	GO:0008150	biological_process	A0A2K3P1V8	GO:0006730	one-carbon metabolic process
P	GO:0008150	biological_process	A0A2K3NMQ3	GO:0006886	intracellular protein transport
P	GO:0008150	biological_process	A0A2K3P6Y1	GO:0006886	intracellular protein transport
P	GO:0008150	biological_process	A0A2K3MUC7	GO:0019510	S-adenosylhomocysteine catabolic process
P	GO:0008150	biological_process	A0A2K3NMQ3	GO:0048268	clathrin coat assembly
P	GO:0008150	biological_process	A0A2K3P6Y1	GO:0048268	clathrin coat assembly
P	GO:0008150	biological_process	A0A2K3NKS8	GO:0006468	protein phosphorylation
P	GO:0008150	biological_process	A0A2K3MA51	GO:0019538	protein metabolic process
P	GO:0008150	biological_process	A0A2K3MUW7	GO:0016310	phosphorylation
P	GO:0008150	biological_process	A0A2K3NKS8	GO:0016310	phosphorylation
P	GO:0008150	biological_process	A0A2K3NRS2	GO:0016310	phosphorylation
P	GO:0008150	biological_process	A0A2K3PJL4	GO:0016310	phosphorylation
P	GO:0008150	biological_process	A0A2K3P8U8	GO:0006811	ion transport
P	GO:0008150	biological_process	A0A2K3PCD9	GO:0006807	nitrogen compound metabolic process
P	GO:0008150	biological_process	A0A2K3P3K7	GO:0009086	methionine biosynthetic process
P	GO:0008150	biological_process	A0A2K3NLX6	GO:0015991	ATP hydrolysis coupled proton transport
P	GO:0008150	biological_process	A0A2K3MW20	GO:1901800	positive regulation of proteasomal protein catabolic process
P	GO:0008150	biological_process	A0A2K3MW20	GO:0030163	protein catabolic process
P	GO:0008150	biological_process	A0A2K3PCD9	GO:0006537	glutamate biosynthetic process
P	GO:0008150	biological_process	A0A2K3NQ11	GO:0006086	acetyl-CoA biosynthetic process from pyruvate
P	GO:0008150	biological_process	A0A2K3NWT6	GO:0006011	UDP-glucose metabolic process
P	GO:0009056	catabolic process	A0A2K3NQ11	GO:0006096	glycolytic process
P	GO:0009056	catabolic process	A0A2K3MUC7	GO:0019510	S-adenosylhomocysteine catabolic process
P	GO:0009056	catabolic process	A0A2K3MW20	GO:1901800	positive regulation of proteasomal protein catabolic process
P	GO:0009056	catabolic process	A0A2K3MW20	GO:0030163	protein catabolic process
P	GO:0009058	biosynthetic process	A0A2K3NMF1	GO:0019264	glycine biosynthetic process from serine
P	GO:0009058	biosynthetic process	A0A2K3P3K7	GO:0008652	cellular amino acid biosynthetic process
P	GO:0009058	biosynthetic process	A0A2K3MUW7	GO:0046940	nucleoside monophosphate phosphorylation
P	GO:0009058	biosynthetic process	A0A2K3P1V8	GO:0006556	S-adenosylmethionine biosynthetic process
P	GO:0009058	biosynthetic process	A0A2K3P3K7	GO:0009086	methionine biosynthetic process
P	GO:0009058	biosynthetic process	A0A2K3PCD9	GO:0006537	glutamate biosynthetic process
P	GO:0009058	biosynthetic process	A0A2K3NQ11	GO:0006086	acetyl-CoA biosynthetic process from pyruvate
P	GO:0016192	vesicle-mediated transport	A0A2K3NMQ3	GO:0016192	vesicle-mediated transport

P	GO:0016192	vesicle-mediated transport	A0A2K3P6Y1	GO:0016192	vesicle-mediated transport
P	GO:0022607	cellular component assembly	A0A2K3NMQ3	GO:0048268	clathrin coat assembly
P	GO:0022607	cellular component assembly	A0A2K3P6Y1	GO:0048268	clathrin coat assembly
P	GO:0034641	cellular nitrogen compound metabolic process	A0A2K3NQ11	GO:0006096	glycolytic process
P	GO:0034641	cellular nitrogen compound metabolic process	A0A2K3MUW7	GO:0006139	nucleobase-containing compound metabolic process
P	GO:0034641	cellular nitrogen compound metabolic process	A0A2K3NMF1	GO:0035999	tetrahydrofolate interconversion
P	GO:0034641	cellular nitrogen compound metabolic process	A0A2K3MUW7	GO:0046940	nucleoside monophosphate phosphorylation
P	GO:0034641	cellular nitrogen compound metabolic process	A0A2K3MUC7	GO:0019510	S-adenosylhomocysteine catabolic process
P	GO:0034641	cellular nitrogen compound metabolic process	A0A2K3NQ11	GO:0006086	acetyl-CoA biosynthetic process from pyruvate
P	GO:0034641	cellular nitrogen compound metabolic process	A0A2K3NWT6	GO:0006011	UDP-glucose metabolic process
P	GO:0034655	nucleobase-containing compound catabolic process	A0A2K3MUC7	GO:0019510	S-adenosylhomocysteine catabolic process
P	GO:0044281	small molecule metabolic process	A0A2K3NQ11	GO:0006096	glycolytic process
P	GO:0044281	small molecule metabolic process	A0A2K3NMF1	GO:0019264	glycine biosynthetic process from serine
P	GO:0044281	small molecule metabolic process	A0A2K3P3K7	GO:0008652	cellular amino acid biosynthetic process
P	GO:0044281	small molecule metabolic process	A0A2K3NMF1	GO:0035999	tetrahydrofolate interconversion
P	GO:0044281	small molecule metabolic process	A0A2K3MUW7	GO:0046940	nucleoside monophosphate phosphorylation
P	GO:0044281	small molecule metabolic process	A0A2K3MUC7	GO:0006730	one-carbon metabolic process
P	GO:0044281	small molecule metabolic process	A0A2K3NMF1	GO:0006730	one-carbon metabolic process
P	GO:0044281	small molecule metabolic process	A0A2K3P1V8	GO:0006730	one-carbon metabolic process
P	GO:0044281	small molecule metabolic process	A0A2K3MUC7	GO:0019510	S-adenosylhomocysteine catabolic process
P	GO:0044281	small molecule metabolic process	A0A2K3P3K7	GO:0009086	methionine biosynthetic process
P	GO:0044281	small molecule metabolic process	A0A2K3PCD9	GO:0006537	glutamate biosynthetic process
P	GO:0044281	small molecule metabolic process	A0A2K3NQ11	GO:0006086	acetyl-CoA biosynthetic process from pyruvate
P	GO:0044281	small molecule metabolic process	A0A2K3NWT6	GO:0006011	UDP-glucose metabolic process
P	GO:0051186	cofactor metabolic process	A0A2K3NMF1	GO:0035999	tetrahydrofolate interconversion

P	GO:0051186	cofactor metabolic process	A0A2K3P1V8	GO:0006556	S-adenosylmethionine biosynthetic process
P	GO:0051186	cofactor metabolic process	A0A2K3MUC7	GO:0019510	S-adenosylhomocysteine catabolic process
P	GO:0051186	cofactor metabolic process	A0A2K3NQ11	GO:0006086	acetyl-CoA biosynthetic process from pyruvate
P	GO:0051276	chromosome organization	A0A2K3NKS8	GO:0016573	histone acetylation
P	GO:0055085	transmembrane transport	A0A2K3NLX6	GO:1902600	proton transmembrane transport
P	GO:0055085	transmembrane transport	A0A2K3P8U8	GO:0120029	proton export across plasma membrane
P	GO:0055085	transmembrane transport	A0A2K3NV46	GO:0055085	transmembrane transport
P	GO:0055085	transmembrane transport	A0A2K3NLX6	GO:0015991	ATP hydrolysis coupled proton transport
P	GO:0065003	protein-containing complex assembly	A0A2K3NMQ3	GO:0048268	clathrin coat assembly
P	GO:0065003	protein-containing complex assembly	A0A2K3P6Y1	GO:0048268	clathrin coat assembly
P	GO:0071554	cell wall organization or biogenesis	A0A2K3PGD6	GO:0071669	plant-type cell wall organization or biogenesis
P	GO:0071554	cell wall organization or biogenesis	A0A2K3PKL4	GO:0071669	plant-type cell wall organization or biogenesis

C - cellular_component; F - molecular_function; P - biological_process
