# Supplementary material for: Composition of Proteins Associated with Red Clover (Trifolium pratense) and the Microbiota Identified in Honey
Source: Life (Basel). 2024 Jul 10;14(7):862. doi: 10.3390/life14070862 (PMC11278118; doi:10.3390/life14070862)
Supplement: Supplementary file 1 [file life-14-00862-s001.zip › Table S2.pdf]

Supplementary Table S2. The number of proteins involved in biological processes of red clover proteins (*Trifolium pratense*) annotated for different honey samples.

Input_GO Identifier	GO Term Name	Number of proteins
GO:0044281	small molecule metabolic process	8
GO:0006412	translation	6
GO:0009058	biosynthetic process	6
GO:0034641	cellular nitrogen compound metabolic process	5
GO:0006520	cellular amino acid metabolic process	4
GO:0006790	sulfur compound metabolic process	4
GO:0006810	transport	4
GO:0051186	cofactor metabolic process	4
GO:0009056	catabolic process	3
GO:0055085	transmembrane transport	3
GO:0005975	carbohydrate metabolic process	2
GO:0006091	generation of precursor metabolites and energy	2
GO:0006464	cellular protein modification process	2
GO:0016192	vesicle-mediated transport	2
GO:0022607	cellular component assembly	2
GO:0065003	protein-containing complex assembly	2
GO:0071554	cell wall organization or biogenesis	2
GO:0006457	protein folding	1
GO:0007165	signal transduction	1
GO:0034655	nucleobase-containing compound catabolic process	1
GO:0051276	chromosome organization	1
