# Supplementary material for: Composition of Proteins Associated with Red Clover (Trifolium pratense) and the Microbiota Identified in Honey
Source: Life (Basel). 2024 Jul 10;14(7):862. doi: 10.3390/life14070862 (PMC11278118; doi:10.3390/life14070862)
Supplement: Supplementary file 1 [file life-14-00862-s001.zip › Table S3.pdf]

Supplementary Table S3. The protein molecular functions annotation of red clover proteins (*Trifolium pratense*) of different honey samples.

Input_GO Identifier	GO Term Name	Number of proteins
GO:0043167	ion binding	17
GO:0003735	structural constituent of ribosome	6
GO:0016491	oxidoreductase activity	5
GO:0016301	kinase activity	4
GO:0016887	ATPase activity	3
GO:0022857	transmembrane transporter activity	3
GO:0008168	methyltransferase activity	2
GO:0016853	isomerase activity	2
GO:0003723	RNA binding	1
GO:0005198	structural molecule activity	1
GO:0008233	peptidase activity	1
GO:0016765	transferase activity, transferring alkyl or aryl (other than methyl) groups	1
GO:0016779	nucleotidyltransferase activity	1
GO:0016798	hydrolase activity, acting on glycosyl bonds	1
GO:0016829	lyase activity	1
GO:0016874	ligase activity	1
GO:0019843	rRNA binding	1
GO:0051082	unfolded protein binding	1
