# Supplementary material for: Composition of Proteins Associated with Red Clover (Trifolium pratense) and the Microbiota Identified in Honey
Source: Life (Basel). 2024 Jul 10;14(7):862. doi: 10.3390/life14070862 (PMC11278118; doi:10.3390/life14070862)
Supplement: Supplementary file 1 [file life-14-00862-s001.zip › Table S4.pdf]

Supplementary Table S4. Cellular components of red clover proteins (*Trifolium pratense*) identified by MS and annotated for different honey samples.

Input_GO Identifier	GO Term Name	Number of reported peptides for protein
GO:0005575	cellular_component	9
GO:0005622	intracellular	6
GO:0005623	cell	6
GO:0005634	nucleus	1
GO:0005654	nucleoplasm	1
GO:0005737	cytoplasm	6
GO:0005739	mitochondrion	1
GO:0005794	Golgi apparatus	1
GO:0005840	ribosome	7
GO:0005886	plasma membrane	2
GO:0031410	cytoplasmic vesicle	1
GO:0032991	protein-containing complex	6
GO:0043226	organelle	5
